# The transcriptional regulatory network of the *Escherichia coli* MG1655 reference strain

**DOI:** 10.1093/nar/gkag059

**Published:** 2026-02-02

**Authors:** Heera Bajpe, Jongoh Shin, Ying Hefner, Richard Szubin, Jaemin Sung, Yuan Yuan, Bernhard O Palsson

**Affiliations:** Department of Bioengineering, University of California San Diego, La Jolla, CA 92093, United States; Department of Bioengineering, University of California San Diego, La Jolla, CA 92093, United States; Department of Bioengineering, University of California San Diego, La Jolla, CA 92093, United States; Department of Bioengineering, University of California San Diego, La Jolla, CA 92093, United States; Department of Bioengineering, University of California San Diego, La Jolla, CA 92093, United States; Department of Bioengineering, University of California San Diego, La Jolla, CA 92093, United States; Department of Bioengineering, University of California San Diego, La Jolla, CA 92093, United States; Department of Pediatrics, University of California San Diego, La Jolla, CA 92093, United States; Department of Bioengineering, Bioinformatics and Systems Biology Program, University of California San Diego, La Jolla, CA 92093, United States; Center for Microbiome Innovation, University of California San Diego, La Jolla, CA 92093, United States; Novo Nordisk Foundation Center for Biosustainability, 2800 Kongens Lyngby, Denmark

## Abstract

The growth of RNA sequencing (RNA-seq) data accompanied by the development of novel scalable data analytic methods has revealed a deep understanding of the composition of bacterial transcriptomes. This new, first-biological-principles understanding has enabled a novel characterization of the function of the transcriptional regulatory network. Here, we present a single-strain wild-type transcriptomic knowledgebase for the model strain *Escherichia coli* MG1655. The associated transcriptomic compendium consists of 584 high-quality RNA-seq samples from wild-type *E. coli* MG1655 generated using a single protocol. These samples range over a wide condition space, including 45 carbon sources and 10 base media. Using independent component analysis, we decomposed the transcriptomic compendium to extract 115 independently modulated sets of genes (iModulons). We find that (i) iModulons explain 75% of variance in the dataset through knowledge enrichment; (ii) 67% of iModulons are associated with single/combined dominant regulators; (iii) iModulon activity profiles of samples can be utilized to elucidate patterns within the transcriptional regulatory network, such as differences in aerobicity; and (iv) the use of transcriptomic data derived from non-wild-type strains results in changes in iModulon gene membership, highlighting the malleability of the transcriptional regulatory network. Altogether, this knowledgebase serves as a resource for multi-scale knowledge mining for transcriptional regulation in *E. coli* MG1655.

## Graphical Abstract

**Graphical Abstract:**
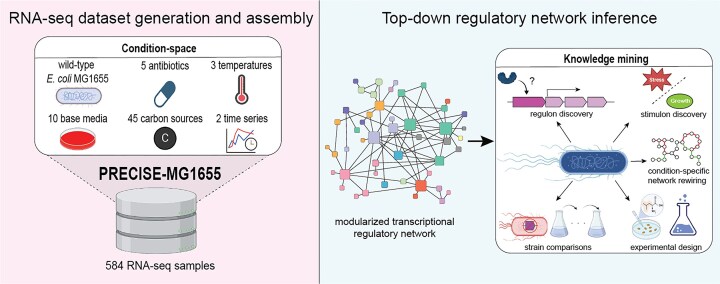

## Introduction

Advancements in sequencing technology and messenger RNA isolation protocols have resulted in a rapid accumulation of RNA sequencing (RNA-seq) data for bacteria. The assembly of large RNA-seq datasets consisting of a wide range of growth conditions has enabled us to probe gene expression in various bacterial species [1, 2]. Importantly, the increase in available RNA-seq data provides the opportunity to build large-scale knowledgebases and data-analytic methods that, together, facilitate the analysis of gene expression and regulation at the genome level [3, 1, 4]. A dataset constructed from various data sources can be tedious and expensive to assemble, while lacking complete metadata and being riddled with batch effects [5, 6]. The compilation of a high-quality, single-protocol dataset would help overcome these challenges.

Such a resource would also further efforts to untangle the complex transcriptional regulatory network (TRN) that governs the ability of an organism to dynamically respond to its environment. While experimental methods, such as chromatin immunoprecipitation assays, are typically employed to define gene-regulator relationships, using bottom-up methods alone may not be feasible in defining the TRN due to its complexity [7]. Along with the availability of large-scale datasets, there is a need for the development of methods that contribute to TRN inference through a top-down fashion.

Independent component analysis (ICA) is a method used to extract statistically independent components from complex datasets. This method was previously found to showcase the best performance in detecting modules of coregulated genes among 42 module detection methods [8] and has proven its utility as a top-down approach to bacterial TRN inference [3, 9]. When applied to bacterial RNA-seq data, ICA extracts independently modulated gene sets, known as iModulons, most of which represent cellular processes and an associated regulator. This method not only modularizes the TRN, but also provides the activity of each iModulon under all growth conditions in the dataset. While ICA provides a valuable and unique knowledge-enriched view of the TRN from a systems standpoint, its application is dependent on the availability of high-quality large-scale datasets representing a wide array of conditions [1].

We previously compiled the PRECISE-1K expression and regulation knowledgebase for the model *Escherichia coli* K-12 strains, MG1655 and BW25113. The expression component of PRECISE-1K is an RNA-seq compendium consisting of 1035 high quality samples generated through a single protocol [1]. With 533 unique conditions, including genetic perturbations and adaptive laboratory evolution (ALE), PRECISE-1K captures both wild-type and non-wild-type mechanisms, allowing for a well-rounded analysis of global expression trends in the two closely related K-12 strains. In addition, the application of ICA to this compendium uncovers a detailed joint iModulon structure for the MG1655 and BW25113 strains, thus forming the regulatory component of the knowledgebase. Our previous studies applying ICA to the PRECISE-1K dataset have demonstrated its potential in knowledge mining, including the identification of new regulons and a better definition of known regulons. Additionally, they have provided new insights into cellular processes that are yet to be fully elucidated [1, 10, 11, 12].

However, the application of ICA in studying strain-specific wild-type regulation against a constant genetic background is yet to be explored. While the TRN structure of different strains of a species overlap, there are regulatory components that are evolutionarily unique to each strain [13]. Our previous study on the PRECISE knowledgebase revealed strain-specific iModulons for the MG1655 and BW25113 strains, suggesting that even minor differences in genetic backgrounds can corrupt our ability to elucidate a pristine TRN for each strain [3]. Hence, there is a need to understand strain-specific regulation in order to uncover the underlying native network that uniquely controls gene expression in each strain. Additionally, samples perturbed through genetic manipulation and ALE result in rewiring of the TRN to accommodate the perturbations [14]. Therefore, the inclusion of such samples in a dataset is not ideal for understanding wild-type regulation. A strain-specific dataset including only wild-type samples would overcome these issues and would also provide a basis for detailing network changes due to perturbations.

Here, we describe an expression and regulation knowledgebase for the wild-type model strain *E. coli* MG1655. The PRECISE-MG1655 knowledgebase contains an expression component consisting of 584 RNA-seq profiles that combine high-quality genetically unperturbed and unevolved samples from PRECISE-1K with newly generated samples, all obtained through a single experimental protocol. Upon the application of ICA, 115 iModulons were extracted that explain a total of 75% of variance in gene expression in the dataset. This iModulon structure forms the regulatory component of the knowledgebase. The variance explained by the iModulons is based on knowledge enrichment; thus, this approach satisfies the requirement of explanatory artificial intelligence and machine learning methods. After modularizing the TRN, we found that 67% of iModulons are linked to single/combined regulators. We also uncovered novel patterns within iModulon gene membership that shed light on independent and interlinked cellular functions. Additionally, we identified groups of iModulons with global activity correlations, revealing functions of the wild-type cell that change in a coordinated manner. Furthermore, we used the iModulon activity spectra to uncover conditions that elicit similar transcriptomic responses related to aerobicity and motility. Moreover, we study the malleability of the TRN by investigating the corruption of iModulon gene membership as a result of the use of samples with mixed genetic background. Finally, using the pure wild-type iModulon structure derived from PRECISE-MG1655, we present a graphical representation for the *E. coli* MG1655 TRN. All data and iModulons from this study can be browsed, analyzed, and downloaded on iModulonDB.org [2].

## Materials and methods

### RNA extraction and library preparation

As previously described [1], 3 ml of cell broth (OD600 ∼ 0.5) was immediately added to two volumes of Qiagen RNA-protect Bacteria Reagent (6 ml). After being vortexed for 5 s, the mixture was incubated at room temperature for 5 min and then centrifuged for 10 min at 11 000 × *g*. The cell pellet obtained after decanting the supernatant was stored at −80°C. After thawing, the cell pellets were incubated with Readylyse Lysozyme, SuperaseIn, Protease K, and 20% sodium dodecyl sulfate for 20 min at 37 °C. Using the Qiagen RNeasy Mini Kit (Cat#74104) columns, total RNA was isolated and purified. Following this, an on-column DNase treatment was performed at room temperature for 30 min. Using a Nanodrop, RNA was quantified, after which quality was evaluated using an RNA-nano chip on a bioanalyzer. All ribosomal RNA (rRNA) was removed using Illumina Ribo-Zero rRNA removal kit (Cat#20037135) for gram-negative bacteria. Sequencing libraries with an average insert length of ∼300 bp were created using a KAPA stranded RNA-Seq Kit (Kapa Biosystems KK8401) by following the manufacturer’s protocol. Libraries were then run on Illumina or Element Biosciences sequencing platforms (listed in sample metadata).

### Data processing and quality control

As previously described, a Nextflow pipeline for processing bacterial RNA-seq data was applied to candidate samples in the dataset using Amazon Web Services [3, 15, 16]. Trim Galore (https://www.bioinformatics.babraham.ac.uk/projects/trim_galore/) was used to perform raw read trimming. The trimmed reads were then assessed using FastQC (https://www.bioinformatics.babraham.ac.uk/projects/fastqc/) and then aligned to the reference genome (RefSeq accession number: NC_000913.3) using Bowtie [17]. Next, RSeQC was used to infer read direction [18], after which read counts were generated using featureCounts [19]. Finally, MultiQC was used to compile all quality control metrics [20]. The expression data were then converted to units of log_2_ transcripts per million (log_2_[TPM]).

To ensure samples are of high quality, those that failed any of the following FastQC metrics were discarded: per_base_sequence_quality, per_sequence_quality_scores, per_base_n_content, and adapter_content. Samples with <500 000 reads mapped to coding sequences were also discarded. Hierarchical clustering was then used to visualize global correlations (Pearson’s *r*) between gene expression (log_2_[TPM]) of all samples, after which outliers having atypical expression profiles were dropped. The metadata of all samples passing these quality metrics was curated, and samples without a biological replicate or Pearson’s *r* < 0.95 between replicate gene expression (log_2_[TPM]) were discarded. After running this workflow, the RNA-seq compendium contained 584 samples (Supplementary Table S1 and Supplementary Note S1). The log_2_[TPM] data were then normalized to the reference condition for each specific project to remove batch effects, with the following exceptions: single-condition projects and miscellaneous projects (“misc” and “misc2”) were normalized to the reference condition of the “control” project (Supplementary Table S2). All relevant files, including metadata, log_2_[TPM], and quality control results can be found on the Zenodo repository for the study.

### Computing iModulons with ICA

ICA was applied to the centered log_2_[TPM] data by running the Scikit-learn [21] algorithm, FastICA [22], as previously described [3, 15]. This algorithm decomposes the input matrix of gene expression profiles (**X**) into an iModulon matrix (**M**) (Supplementary Table S3) and an activity matrix (**A**) (Supplementary Table S4). The **M** matrix contains the weight of each gene in all components extracted by ICA, whereas the **A** matrix contains the activity of each component across all samples in the compendium.

As the ICA algorithm requires the number of components (dimensionality) to be specified, we selected an optimal dimensionality using the OptICA method as previously described [23]. The optimal dimensionality selected for the PRECISE-MG1655 dataset was 190, with the ICA run extracting 115 iModulons.

The **M** matrix specifies the association of each gene with a particular iModulon, with higher weightings representing a greater association. iModulons are defined by applying a threshold that allows genes to be segregated based on their weights in an iModulon. As previously done, these thresholds were calculated for each iModulon using the D’Augustino’s *K*_2_ test for normality [3]. The gene membership of the 115 iModulons was thus determined based on the calculated cutoffs. The binarized **M** matrix (Supplementary Table S5) provides the gene membership of each iModulon.

### iModulon annotation and curation

Gold standard TRN annotations from RegulonDB [24] were downloaded and used to compute enrichment of genes in each iModulon against regulons defined on RegulonDB through Fisher’s Exact Test [Benjamini-Hochberg false discovery rate (FDR) = 10^−5^]. Assessing and expanding regulator enrichment for iModulons was done as previously described [1]. All details regarding regulator enrichment, including statistics and evidence levels selected for enrichment, are listed in the iModulon table (Supplementary Table S6). iModulons with significant enrichment for a single or combination of regulators were annotated as “Regulatory” iModulons. Those lacking significant enrichment for a regulator were categorized as follows: “Biological” for iModulons with a known biological function; “Technical” for iModulons with a single highly weighted gene; “Uncharacterized” for iModulons with unknown function.

### Differential iModulon activation

Differential iModulon activities between two conditions or sets of conditions were calculated as previously described [3]. The statistical significance of the differences in iModulon activation were determined by comparing the absolute value of the difference in mean iModulon activities between the conditions and the log-normal distribution of the iModulon. iModulons with a threshold >5 and FDR less than the selected value were considered to be differentially activated.

## Results

### PRECISE-MG1655 is a high-quality strain-specific knowledgebase for wild-type *E. coli* MG1655

**Figure 1.**
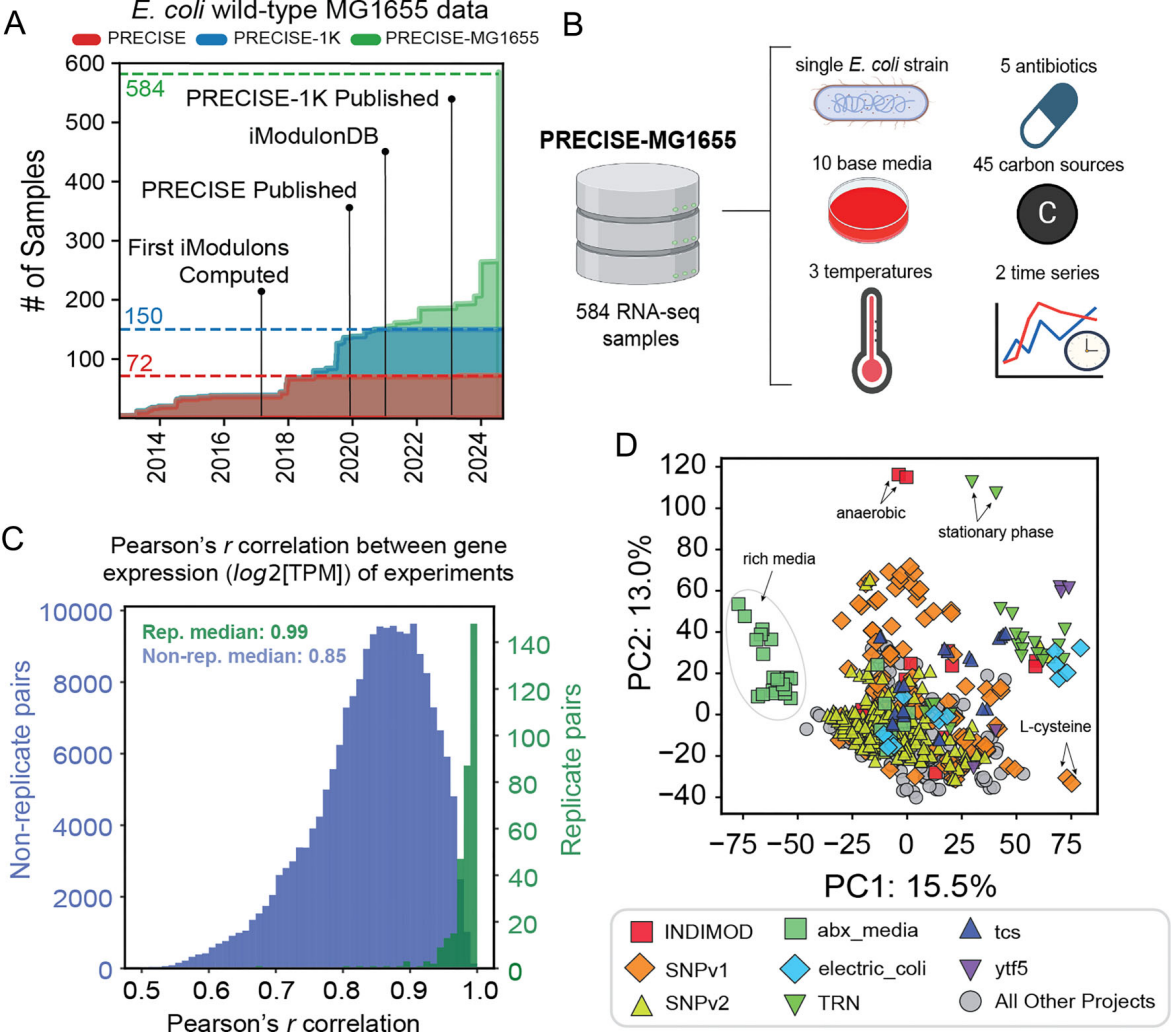
Overview of the PRECISE-MG1655 dataset. (**A**) Growth of single-protocol RNA-seq data for wild-type *E. coli* MG1655 present in the PRECISE, PRECISE-1K, and PRECISE-MG1655 datasets. (**B**) Visual representation of the composition of the condition-space of the PRECISE-MG1655 dataset based on select metadata categories [created in BioRender. Palsson, B. (2026) https://BioRender.com/w6f4azs]. (**C**) Histogram of Pearson’s *r* correlation between gene expression (log_2_[TPM]) of all samples in the dataset, including both replicate (green bars) and non-replicate (blue bars) pairs. (**D**) Scatter plot of the first two principal components (PCs) in the dataset, colored by project (*n* = 584). The metadata for each project is listed in Supplementary Table S1.

We constructed the PRECISE-MG1655 knowledgebase to enable the study of gene expression and regulation in wild-type *E. coli* MG1655. The MG1655 strain is one of the best characterized strains of the *E. coli* species, which is the most studied bacterial species, representing ~21% of peer-reviewed literature on bacteria [25]. The expression component of PRECISE-MG1655 is a single-strain, single-protocol RNA-seq compendium consisting of 584 wild-type samples generated by a single research group employing a standardized protocol for experimentation and data processing. The collection of samples includes 150 genetically unperturbed and unevolved samples from the previous PRECISE-1K dataset. In addition, the dataset contains 434 wild-type samples that were generated after the publication of PRECISE-1K (Fig. 1A and Supplementary Table S10). The 33 projects from which this collection of samples originates contain a variety of conditions, including 45 carbon sources, 10 base media types, 5 antibiotics, 3 temperatures, 2 time series, and different growth phases (Fig. 1B and Supplementary Table S1). This genetically pristine transcriptomic compendium is of high quality, with a median Pearson’s *r* correlation of 0.99 between gene expression (log_2_[TPM]) of replicates (Fig. 1C). Thus, the PRECISE-MG1655 dataset is the largest high-precision single-protocol dataset for wild-type *E. coli* MG1655 and empowers the study of its native TRN.

We leveraged the diverse condition space of the PRECISE-MG1655 dataset to study genome-scale patterns within the expression of genes and the expression profiles of RNA-seq samples (Supplementary Fig. S1 and Supplementary Note S2). Principal component analysis (PCA) revealed that the separation between samples is largely due to growth conditions (Fig. 1D). The first two PCs together explain 28.5% of variance in gene expression in the data, with a total of 180 components required to explain 95% of the variance (Supplementary Fig. S2). We also clustered gene expression (log_2_[TPM]) correlations, as well as the correlation of expression profiles of samples in the dataset (Supplementary Fig. S3), to reveal patterns within the gene expression matrix, details of which can be found in Supplementary Note S3. Our findings from these analyses suggest that there exists an underlying structure to the TRN that needs biological explanation. To unveil this complex structure and gain deeper insights into the TRN, we applied our ICA algorithm to the PRECISE-MG1655 dataset.

### ICA uncovers a genome-scale characterization of variation in the *E. coli* MG1655 transcriptome

The application of our ICA algorithm to the PRECISE-MG1655 dataset resulted in the extraction of 115 iModulons (Supplementary Fig. S4 and Supplementary Tables S5 and S6; see “Methods” section) [15]. Each iModulon represents a group of genes that are co-regulated under conditions within the condition-space of the RNA-seq compendium. This algorithm not only reveals the gene membership of each iModulon (weightings in the columns of the **M** matrix; Supplementary Table S3), but also provides the activities of each iModulon across all conditions compared to the baseline condition for each project (activities in the rows of the **A** matrix; Supplementary Tables S2 and S4). The 115 iModulons explain 75% of variance in gene expression in the dataset. Unlike PCA, the components extracted by ICA have both statistical and biological significance [8, 26, 27]. They yield a knowledge-based explanation for the variation in the PRECISE-MG1655 dataset (Supplementary Fig. S5 and Supplementary Note S4).

**Figure 2.**
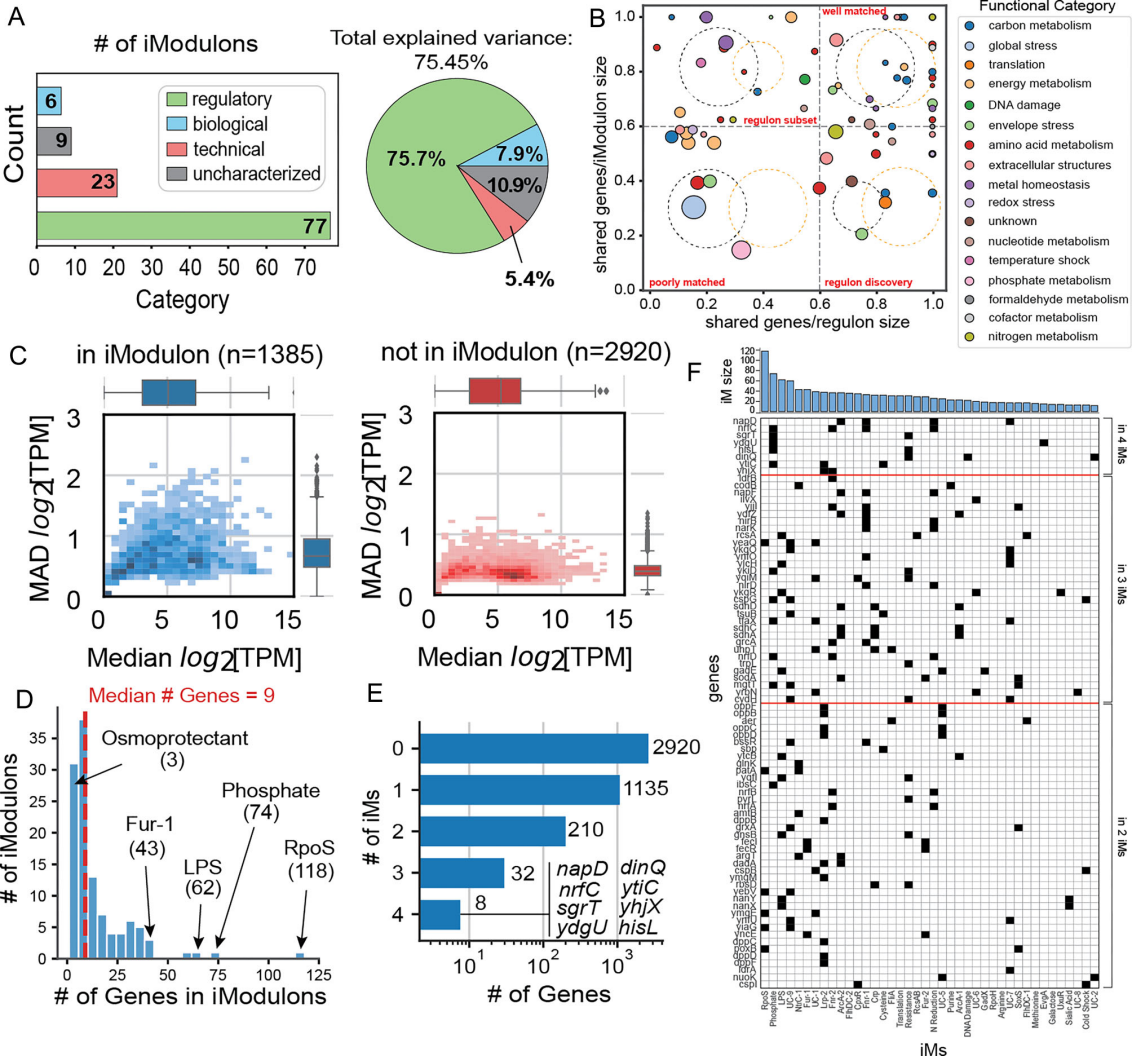
Summary of iModulon structure of PRECISE-MG1655. (**A**) iModulons categorized based on regulator enrichment categories. (**B**) Precision versus recall plot for regulatory iModulons in PRECISE-MG1655. The dashed Venn diagrams depict the type of overlap between regulons (black) and iModulons (orange). The legend specifies the functional category of each iModulon in the plot. (**C**) 2D histogram of median gene expression plotted against median absolute deviation of gene expression for genes in at least one iModulon and those not in any iModulon. (**D**) Histogram of iModulon counts based on the number of genes in each iModulon. (**E**) Histogram of gene counts based on multi-iModulon membership. (**F**) Top 10% of entries in gene presence/absence plot (40 iModulons/80 genes), with genes sorted by iModulon membership count and iModulons sorted by size in descending order. The bar chart represents the size of each iModulon. (iM: iModulon)

After curation and characterization of the iModulons, they were categorized into four groups based on regulator enrichment: (i) Regulatory, (ii) Biological, (iii) Uncharacterized, and (iv) Technical (Fig. 2A). The largest category of iModulons, “Regulatory,” consists of 77 iModulons that are significantly enriched for genes in a single regulon or a combination of regulons (Supplementary Fig. S6; see “Methods” section). This category of Regulatory iModulons explains 76% of the total variance in gene expression captured by the PRECISE-MG1655 dataset. As found with PRECISE-1K, the precision (fraction of iModulon genes captured by the enriched regulon) and recall (fraction of genes in enriched regulon found within iModulon) of iModulons capturing smaller regulons tends to be higher compared to those capturing larger regulons (Fig. 2B) [1]. Cases of low similarity between genes found in regulatory iModulons and their enriched regulons may be due to an iModulon capturing a subset of more strongly regulated genes within the enriched regulon. However, condition-specific activation of subsets of larger regulons and indirect transcription factor interactions may also play a role in low similarity. Further comparisons of iModulon and regulon gene membership can be performed using iModulonDB (Supplementary Note S5). The six “Biological” iModulons, linked to a specific biological function based on the functional annotation of gene members (rather than being significantly enriched for a regulon), explain 8% of the total variance, whereas the nine “Uncharacterized” iModulons that represent unknown cellular functions explain 11% of the total variance. The remaining 5% of the total variance is captured by the 23 “Technical” iModulons that contain a single highly weighted gene. These iModulons likely capture noise in the expression of these genes.

While not being expressed at a significantly higher level (*P* = .12), genes within iModulons have a significantly higher median absolute deviation compared to those not found in any iModulon (*P* = 1.340165E-265, Mann–Whitney *U* test, *m* = 1385, *n* = 2920) [1]. We observed that 32.17% of genes (1385/4305) were members of at least one iModulon (Fig. 2C). These 1385 genes include those belonging to the previously defined shallow (1180 partially characterized genes) and deep (719 uncharacterized genes) y-ome [26], with 28.1% (331/1180) and 31.6% (227/719) iModulon gene coverage within the compendium, respectively (Supplementary Fig. S7A and B).

We also categorized iModulon genes based on their presence in the core, accessory, and rare genome of the *E. coli* pangenome [27]. Each category had an iModulon gene coverage of 31.3% (705/2255), 32.5% (506/1557), and 10% (1/10), respectively. The presence of y-ome, accessory, and rare genes that are not fully characterized within iModulons showcases the potential of iModulon analysis in furthering gene function annotations (Supplementary Fig. S7C). However, there is a need for more diverse growth conditions for wild-type *E. coli* MG1655 that would enable a higher iModulon gene coverage.

### iModulon gene membership is largely independent, with few multi-iModulon genes

Further analysis of iModulon composition using the **M** matrix allows us to gain deeper insight into the modular representation of the TRN. The largest iModulon extracted, RpoS, contains 118 genes and is enriched for the global regulator RpoS, while the smallest non-technical iModulon is the Osmoprotectant iModulon, containing three genes involved in the specialized function of glycine-betaine osmolyte production (Fig. 2D). The median iModulon size is nine genes. While 82% of genes in iModulons (1135/1385) belong to only a single iModulon, the remaining genes are members of up to four iModulons (Fig. 2E and Supplementary Fig. S8A). The multi-iModulon gene count of 250 in PRECISE-MG1655 is lower compared to that in PRECISE-1K, which contains 879 multi-iModulon genes that are found in up to seven iModulons. The lower multi-iModulon gene count in PRECISE-MG1655 may indicate a better separation of statistically independent signals detected within the transcriptome by ICA as a result of the “purified” single-strain wild-type dataset.

Of the 250 multi-iModulon genes in PRECISE-MG1655, most genes (210/250) are members of just two iModulons. However, 32 genes are members of three iModulons, and eight genes are members of four iModulons. The iModulons to which this set of 40 (i.e. 32 + 8) genes belong are often large in gene count (Fig. 2F and Supplementary Fig. S8B). The Phosphate and Fnr-1 iModulons contain the highest number of multi-iModulon genes that are in more than two iModulons, specifically 10 genes each (Supplementary Fig. S8C). Interestingly, several multi-iModulon genes present in more than two iModulons belong to those that are involved in cellular respiration, namely Fnr-1, Fnr-2, ArcA-1, ArcA-2, and N Reduction. For example, the genes *napDF* are members of the ArcA-2, Fnr-1, and N Reduction iModulons. The presence of genes found in multiple iModulons that fall under the same functional category represents the complexity and interconnectedness between these independent signals.

### Activity state of the modularized TRN

While the iModulon components obtained from the **M** matrix lay out a structural representation for the TRN, the **A** matrix provides the activity state of the modularized TRN across the entire condition space of the compendium. A close analysis of the rows of the **A** matrix enables us to study and compare iModulon activating conditions (Supplementary Fig. S9A and Supplementary Note S6). Clustering iModulons based on their activities across the condition-space of the compendium reveals patterns that highlight relationships between the iModulons. Similarities between iModulon activities may be prevalent across the entire dataset, as seen with the UC-3 and Translation iModulons (Supplementary Fig. S9B). On the other hand, these similarities may be more pronounced in a subset of samples. For instance, the similarity between several iModulons involved in the assembly of extracellular structures (FliA, FlhDC-2, LPS, and UC-1 iModulons) can be attributed to activities in samples belonging to the “SNPv1” project. Hence, relationships between iModulons may be global or condition-specific.

### Groups of iModulons with correlated activities form stimulons

We then clustered iModulons based on their correlated activities, enabling the identification of groups of iModulons with coordinated activities across the compendium (Supplementary Fig. S10). These clusters, referred to as stimulons, provide a unique lens through which the underlying structure of the TRN can be studied based on its activity state [28]. While each iModulon represents an independent transcriptional signal, their activities can be highly similar with the exception of a few conditions. iModulons with high activity correlation, but no shared genes, tend to represent coordinated actions between individual cellular functions.

**Figure 3.**
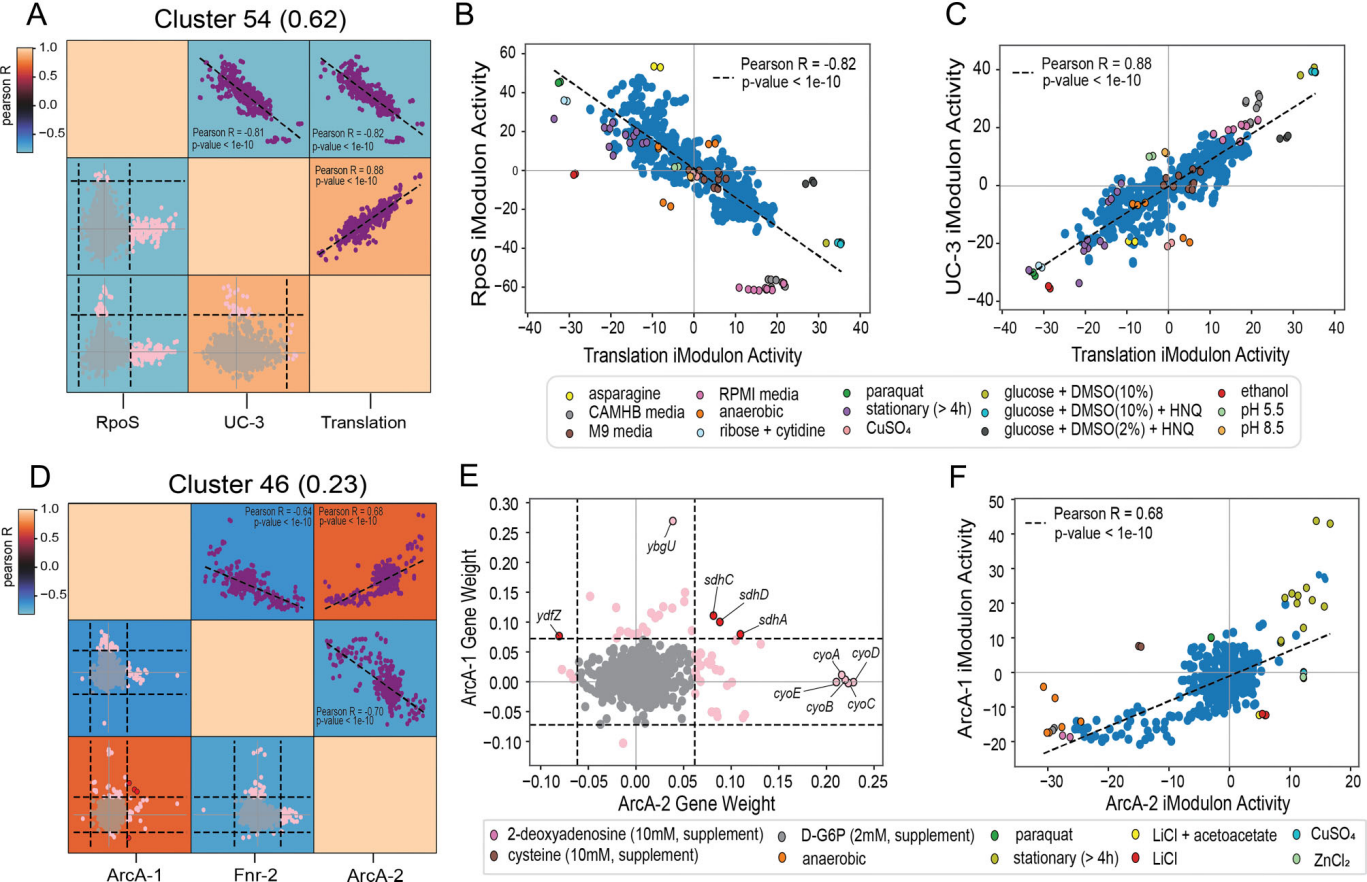
Rows of the **A** matrix. (**A**) Fear-greed stimulon extracted from clustermap of Pearson’s *r* correlation of iModulon activities (Supplementary Fig. S10), consisting of the RpoS, UC-3, and Translation iModulons. Scatter plots in purple show the correlation between the activities of each pair of iModulons. Scatter plots in gray/pink show differential iModulon membership (DiMM) plots for each pair of iModulons. Dotted lines represent the gene weight threshold for each iModulon. Pink and gray dots are used to depict whether a gene is present in only one of the iModulons or does not belong to either iModulon, respectively. Activity phase planes for pairs of iModulons: Translation and RpoS iModulons (**B**), Translation and UC-3 iModulons (**C**). Select conditions have been highlighted and described in the legend, with sample details listed in Supplementary Table S7. (**D**) Cellular respiration stimulon extracted from clustermap, consisting of the ArcA-1, Fnr-2, and ArcA-2 iModulons. Scatter plots in purple show the correlation between the activities of each pair of iModulons. Scatter plots in gray/pink show DiMM plots for each pair of iModulons. Dotted lines represent the gene weight threshold for each iModulon. Red, pink, and gray dots are used to depict whether a gene is shared by both iModulons, present in only one of the iModulons, or does not belong to either iModulon, respectively. (**E**) DiMM plot comparing the weights of each gene in the ArcA-2 and ArcA-1 iModulons. (**F**) Activity phase plane of ArcA-2 and ArcA-1 iModulons. Select conditions have been highlighted and described in the legend, with sample details listed in Supplementary Table S8.

We identified a cluster representing the fear-greed trade-off [29], consisting of the RpoS, Translation, and UC-3 iModulons (Fig. 3A). These iModulons have highly coordinated activities but do not share any genes. The first two are the primary iModulons involved in this trade-off. Specifically, upregulation of the RpoS iModulon coordinated with the downregulation of the Translation iModulon represents responsiveness to stress (“fear”), whereas upregulation of the Translation iModulon coordinated with the downregulation of the RpoS iModulon represents faster growth (“greed”). As expected, the activities of the two iModulons are strongly negatively correlated (Fig. 3B). Conditions that elicit a “fear” response include stationary growth phase, and treatment with oxidative stress-triggering paraquat. On the other hand, conditions exhibiting a “greedy” response include those grown in rich media such as CAMHB and RPMI. Conditions deviating from this trend include the addition of supplements known to disrupt RpoS activity, such as asparagine [30].

The third iModulon in the fear-greed stimulon, UC-3, represents a subset of the regulon of the stringent response regulator, ppGpp. The activity of the UC-3 iModulon has a strong positive correlation with that of the Translation iModulon, in agreement with ppGpp previously being identified as an additional translation-associated dimension of the fear-greed trade-off (Fig. 3C) [29]. Aside from its relation to translation, the exact function of this subset of the ppGpp regulon is unknown. Analysis of the few conditions deviating from the general trend with the primary translation signal may help in further characterizing the UC-3 iModulon. For example, this iModulon is uniquely upregulated in conditions with altered pH. Genes within the iModulon, such as *lysP* and *ydiY*, are linked to pH homeostasis [31, 32]. While further analyses may reveal the function of the UC-3 iModulon, we hypothesize that it represents a subset of ppGpp-regulated genes that are involved in translation as well as other independent functions, such as response to pH fluctuations. In this manner, stimulons help identify evolutionarily linked iModulons through their activity patterns.

### Correlated activities among iModulons highlight complexities of certain cellular functions

Correlated activities among groups of iModulons may also shed light on nuances in cellular functions. iModulons with shared genes, regulators, and correlated activities are often involved in similar functions, with the shared genes providing a genetic basis for this similarity. We identified a second stimulon containing the Fnr-2, ArcA-1, and ArcA-2 iModulons, all three of which are involved in cellular respiration (Fig. 3D). Taking a closer look at the ArcA-1 and ArcA-2 iModulons, each represents a subset of the ArcA regulon involved in modulating cellular energetics based on the redox state of the cell; the ArcA-2 iModulon contains subunits of cytochrome bo3 (*cyoABCDE*) and several genes playing a role in the tricarboxylic acid cycle, while the ArcA-1 iModulon consists of genes that are involved in other aerobic metabolism pathways, such as fatty acid and glycolate metabolism [33, 34]. The two iModulons share four genes: *sdhACD* and *ydfZ* (Fig. 3E). Considering the important role played by succinate dehydrogenase in energy production, it is likely that the*sdhACD* genes are key targets in energy dynamics mediated by ArcA [35].

Accounting for overlapping gene members, regulators, and involvement in similar pathways, it is anticipated that the activities of the two iModulons are strongly correlated (Fig. 3F). As expected, both iModulons are upregulated in stationary-phase samples and downregulated in anaerobic conditions. The difference in the extent to which each iModulon is activated reveals the intricacies in condition-specific control of energy production. On the other hand, divergence from this trend can be attributed to regulation associated with the dominant gene members. For instance, cysteine-treated samples display a strong downregulation of the ArcA-1 iModulon only. The breakdown products of cysteine metabolism include H_2_S, which represses cytochrome bo3 while leaving other terminal oxidases unaffected [36, 37, 38]. The highly weighted genes of the ArcA-1 iModulon are those encoding subunits of cytochrome bo3. Hence, the activity of only ArcA-1 is strongly affected by cysteine supplementation. iModulons with shared genes, regulators, and correlated activities thus highlight the highly complex nature of dynamic functions, such as respiration, that are crucial to survival.

### Correlated columns in A reveal conditions generating similar transcriptomic responses

**Figure 4.**
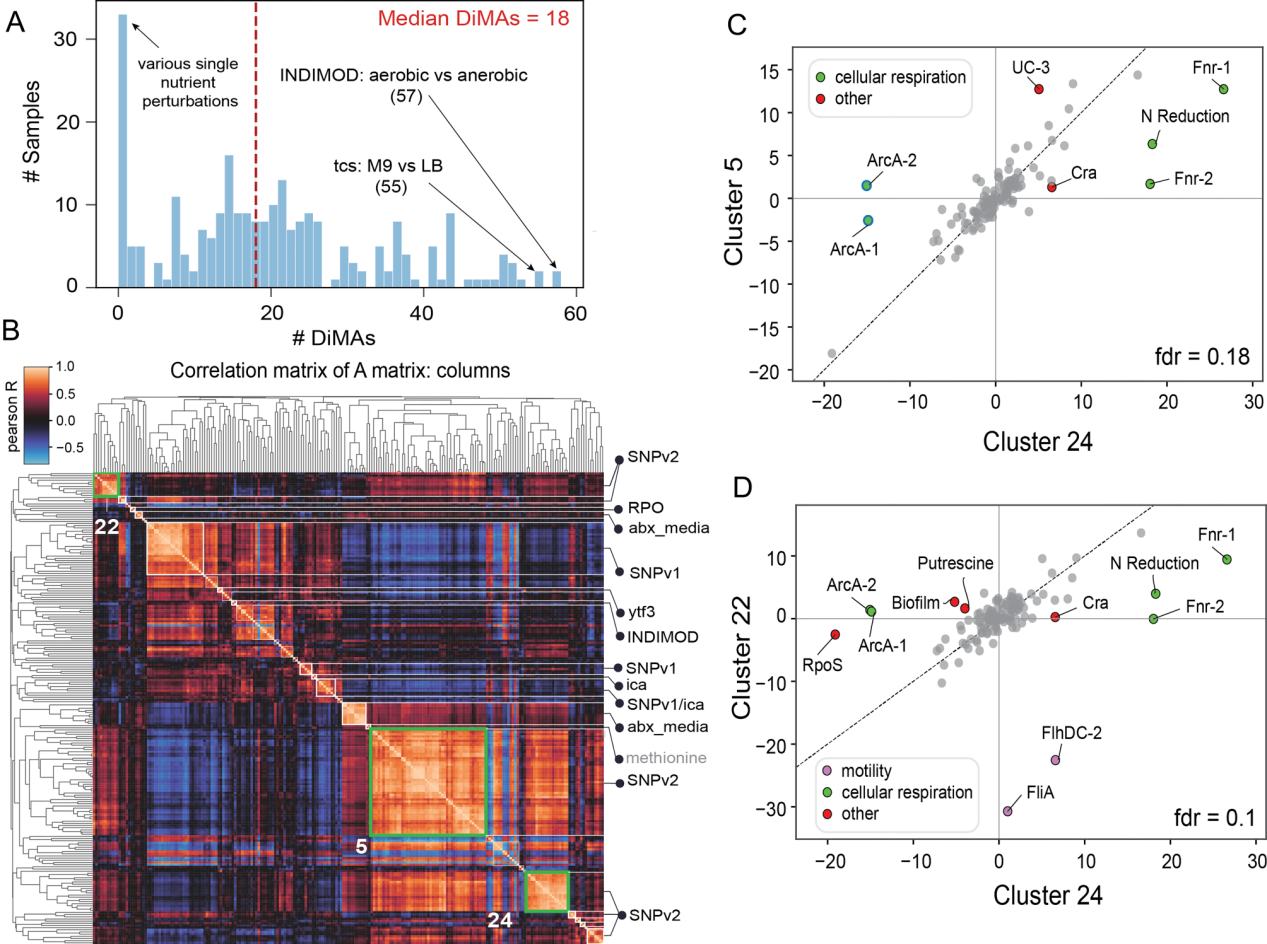
iModulon activity profiles of select samples.(**A**) Histogram of the number of differentially activated iModulons in pairwise comparison of project-specific control and test samples. All projects with an in-project reference used as the project baseline condition have been included. Highlighted projects are formatted as “project code: condition 1 versus condition 2.” (DiMAs: differential iModulon activations). (**B**) Clustermap of Pearson’s *r* correlation of sample activity profiles for select samples. Project-specific clusters are labeled in black text, while clusters where samples do not cluster based on project are labeled in gray text. Clusters of interest (clusters 22, 5, and 24) are highlighted in green boxed regions. Mean iModulon activity across all replicates was used. (**C, D**) Differential iModulon activity plot for samples in Cluster 24 versus Cluster 5, and Cluster 24 versus Cluster 22, respectively. Differentially activated iModulons are colored based on their functional category.

The columns of the **A** matrix, containing the iModulon activities of each sample across the compendium, provide a unique perspective to study and compare the genome-wide transcriptomic responses of the bacterium in various conditions. Compared to the baseline condition for each project, we found that the median number of differentially activated iModulons in non-control samples within the same project was 18 (Fig. 4A). Some comparisons between these samples resulted in a notably high number of differentially activated iModulons, such as the comparison of samples grown in anaerobic conditions to those grown at the baseline aerobic conditions (57 iModulons). On the other hand, no significant difference in activities was observed while comparing samples treated with carbon sources like rhamnose to untreated samples.

Clustering the columns of the **A** matrix reveals groups of samples that have similar activity profiles across all iModulons (Supplementary Fig. S9B). We observed that several clusters are project-specific, with some projects corresponding to multiple clusters. For instance, the “abx_media” project, which contains samples grown in different media and treated with different antibiotics, largely splits into clusters based on whether minimal (M9) or rich (CAMBH and RPMI) media was used. However, samples across projects clustered together in some cases; one cluster contained samples treated with reactive oxygen species (ROS)-generating agents (paraquat and 2,2′-dipyridyl) from three different projects, all having similar profiles across the compendium.

Similarly, clustering the activity profile correlations of all samples emphasizes a clear demarcation of samples based on their projects (Fig. 4B and Supplementary Fig. S11). Several projects such as “SNPv2,” containing samples supplemented with different nutrients, split into several clusters, thereby showcasing patterns of interest within the project. Three moderately sized clusters solely consist of samples from the “SNPv2” project: clusters 24, 22, and 5 (Supplementary Fig. S11C–E). Comparison of samples in the three clusters reveal that five iModulons involved in cellular respiration (ArcA-1, ArcA-2, Fnr-1, Fnr-2, and N Reduction) are uniquely differentially activated in cluster 24 (Fig. 4C and D). The activation states of these iModulons clearly indicate that the nutrient supplements in cluster 24 result in a shift in the redox state of the cell. Although the samples were generated in aerobic conditions, the addition of these supplements at the specified concentrations appear to create an environment that favors aerobic pathways being less active and anaerobic pathways being more active compared to the baseline condition of growth in M9-glucose media. This phenomenon may be a result of the cell combining multiple metabolic pathways to optimize growth based on environmental conditions, such as carbon source availability [39]. Alternatively, considering that several of these supplements, such as sorbitol, are fermentable, these observations may be due to carbon overflow mechanisms, resulting in fermentative pathways being activated to optimize proteome allocation [40, 41, 42].

Additionally, we found that another major driver of the segregation between the three clusters is motility, with the FliA and FlhDC-2 iModulons being differentially downregulated in cluster 22 (Fig. 4D and Supplementary Fig. S12). Supplements in cluster 22 include various amino acids, such as serine, valine, leucine, and tryptophan. *Escherichia coli* strains have been found to not be attracted toward the latter three, which likely explains the differentially downregulated activities of the FliA and FlhDC-2 iModulons [43]. However, the bacterium is known to be strongly attracted toward serine [44]. We observed that while samples supplemented with 2mM serine belong to cluster 5 with higher motility, samples with higher concentration (10 mM) belong to cluster 22 with lower motility. It is possible that chemotaxis is reduced at higher concentrations due to previously reported imprecise adaptive chemotactic behavior in the presence of higher levels of attractants [45]. Altogether, the **A** matrix provides a unique perspective for understanding how transcriptomic responses compare across groups of conditions and identifying transcriptomic patterns within the dataset.

### Knowledge mining through cross-knowledgebase comparison

**Figure 5.**
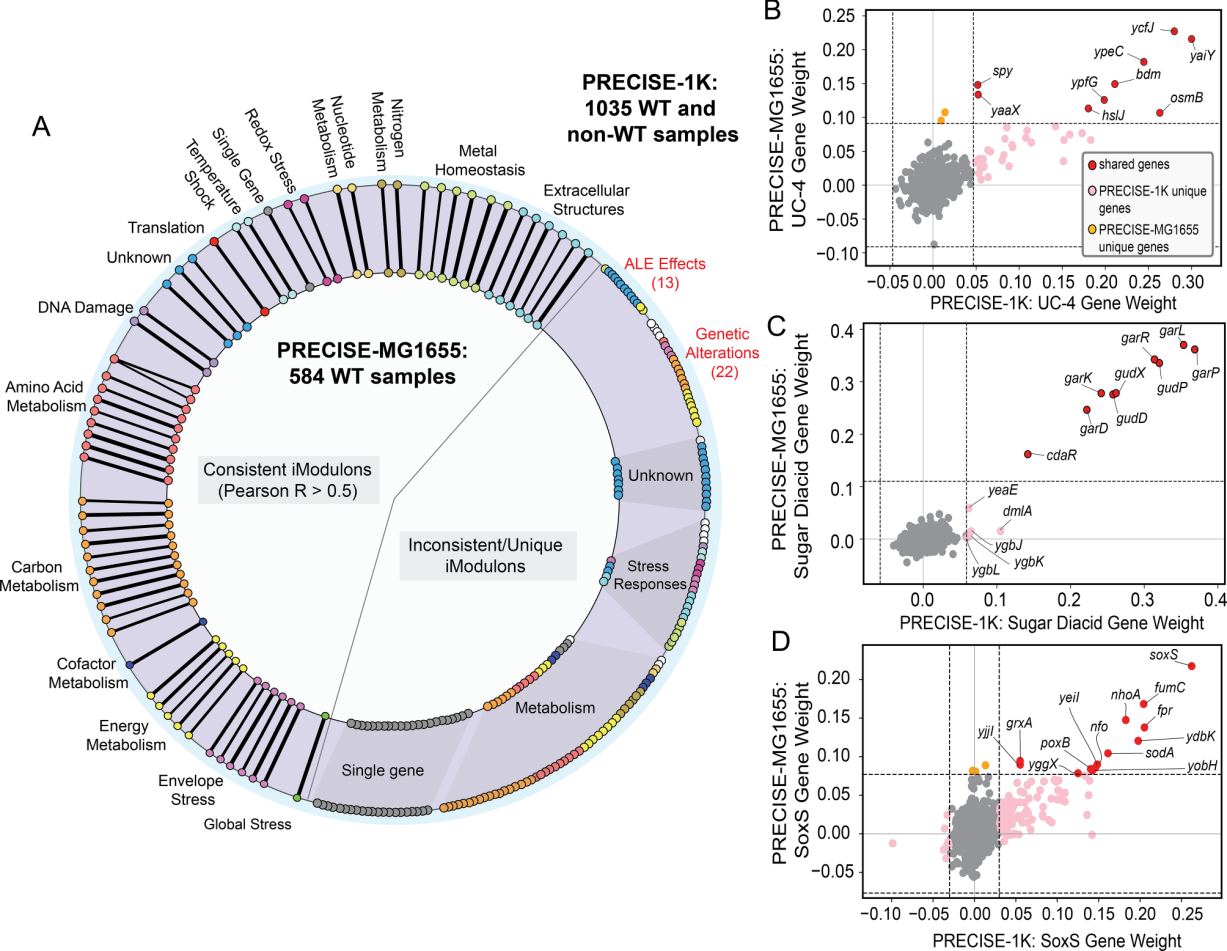
Insights into the *E. coli* MG1655 TRN through cross-knowledgebase iModulon analysis.(**A**) Comparison map of iModulons obtained from PRECISE-1K (outer circle) and PRECISE-MG1655 (inner circle). iModulons are colored based on their functional categories. White circles represent additional unrepresented categories. (**B**–**D**) DiMM plot for the UC-4 iModulons, Sugar Diacid iModulons, and SoxS iModulons of PRECISE-1K and PRECISE-MG1655. Red, pink, orange, and gray dots are used to depict whether a gene is shared by both iModulons, present in only the PRECISE-1K iModulon, present in only the PRECISE-MG1655 iModulon, or does not belong to either iModulon, respectively. (WT: wild-type; ALE: adaptive laboratory evolution)

The PRECISE-MG1655 knowledgebase not only enables the study of wild-type regulation in *E. coli* MG1655, but also enables comparison with non-wild-type knowledgebases to provide insight into changes in TRN structure as a result of genetic perturbations. Genetic manipulation or adaptations through laboratory evolution result in the rewiring of the wild-type TRN to attain homeostasis [14]. The PRECISE-1K dataset contains 885 non-wild-type *E. coli* samples, with a large portion being *E. coli* MG1655 samples perturbed through ALE or genetic manipulation. By comparing iModulons and their gene composition across PRECISE-1K and PRECISE-MG1655, we can gain insight into the regulatory changes that occur to account for these perturbations, and regulatory components that remain consistent. Doing so, we found that 53% of iModulons (61) in PRECISE-MG1655 have a match in PRECISE-1K (Pearson’s *r* > 0.5; Fig. 5A; Supplementary Fig. S13; Supplementary Table S9). Most functional categories of iModulons in PRECISE-MG1655 have a large portion of iModulon matches in PRECISE-1K, including larger categories such as carbon metabolism (11/17) and amino acid metabolism (10/15). While the presence of unmatched iModulons in PRECISE-1K may be partially due to the larger sample collection of the dataset, a notable portion of unmatched iModulons in PRECISE-1K fall under two categories that represent non-wild-type regulation: “ALE effects” [13] and “genetic alterations” [22]. The presence of these iModulons clearly showcases the effects perturbed samples have on TRN inference, and emphasizes a need for precaution while compiling datasets for the study of wild-type gene regulation.

Aside from the “single gene” category, which represents noise in gene expression, the “uncharacterized” category of PRECISE-MG1655 has the highest number of unmatched iModulons. While uncharacterized iModulons may be attributed to noise, they can also represent novel regulatory signals. Interestingly, there were three uncharacterized iModulons that had matches in PRECISE-1K. These iModulons represent unknown signals that are consistently extracted by ICA across datasets and potentially represent transcriptional signals with uncharacterized functions. These iModulons offer an opportunity for discovery of novel signals, as detailed in a case study in Supplementary Note S7.

### Cross-knowledgebase comparisons empower elucidation of the effects of perturbation on the transcriptome

Comparison of the gene membership of consistent iModulons across the two knowledgebases can aid in understanding TRN adaptations as a result of genetic perturbations, such as those resulting from ALE. The UC-4 iModulon, involved in an uncharacterized cell envelope stress response (Supplementary Note S7), is an example of a consistent iModulon (Pearson’s *r* = 0.58) with nine consistent gene members across PRECISE-MG1655 and PRECISE-1K (Fig. 5B). It is of note that the PRECISE-1K UC-4 iModulon contains 30 unique genes. Interestingly, we found that samples from a PRECISE-1K ALE project, “CCK_ptsHIcrr,” uniquely upregulate the UC-4 iModulon as well as the expression of these 30 genes (Supplementary Fig. S15). This study involved the deletion of the phosphotransferase system in a pre-optimized *E. coli* MG1655 strain, followed by evolution to improve glucose uptake and growth phenotype [46]. A key growth improvement in the evolved strain was that it was able to overcome abnormalities in peptidoglycan synthesis by preventing the overactivation of the Rcs phosphorelay system, which is linked to the cell envelope stress response. With the UC-4 iModulon of PRECISE-MG1655 being linked to cell envelope stress, it is likely that the presence of the unique genes in the UC-4 iModulon of PRECISE-1K reflects network changes caused by the strain evolving to overcome envelope stress that is experienced by the unevolved strain.

The Sugar Diacid iModulons are another example of two highly correlated iModulons (Pearson’s *r* = 0.83) in the two knowledgebases, sharing nine genes that are involved in the metabolism of the sugar diacids, galactarate and glutarate (Fig. 5C). However, the iModulon in PRECISE-1K contains five unique genes: *ygbJKL, dmlA*, and *yeaE*. We found that a subset of samples from an ALE project called “Enzyme Promiscuity,” involving the laboratory evolution of *E. coli* MG1655 with the non-native substrate m-tartrate, upregulates both the Sugar Diacid iModulon and expression of the five unique genes (Supplementary Fig. S16 and Supplementary Note S8). The study that generated the samples found that a mutation in the regulator, *ygbI*, during evolution on m-tartrate results in the overexpression of genes including *ygbJKL* [47]. They hypothesized that these genes may have promiscuous activity in m-tartrate metabolism. While further studies would be required to gain a better understanding of the mechanism of m-tartrate metabolism through evolution, our analysis suggests that the inclusion of the five unique gene members of the PRECISE-1K Sugar Diacid iModulon reflects TRN adaptations that enable *E. coli* MG1655 to metabolize m-tartrate.

While these case studies provide insight into TRN adaptations caused by genetic perturbations, they also reveal that the use of non-wild-type samples often result in the inclusion of genes with very low weightings within iModulons. The SoxS iModulons of the two knowledgebases are two consistent iModulons (Pearson’s *r* = 0.56) that largely differ in gene membership due to the inclusion of a large number of low weight genes within the PRECISE-1K iModulon (Fig. 5D). The PRECISE-1K SoxS iModulon contains a total of 104 unique genes. The expression of these genes and the activity of the SoxS iModulon are both upregulated in the ALE project “ROS-TALE,” in which a pre-optimized *E. coli* MG1655 strain was evolved to tolerate high levels of ROS-generating paraquat [10] (Supplementary Fig. S17). This study observed that the TRN adaptations for ROS stress tolerance include transcription factor mutations. Minor regulatory changes caused by these mutations may result in shifts in iModulon gene weights, resulting in the inclusion of low-weight genes within iModulons. This shows the impact of small regulatory adaptations on the purity of iModulon gene membership in comparison to iModulons generated using only wild-type samples.

Together, these case studies highlight the use of the PRECISE-MG1655 knowledgebase in furthering our understanding of how regulatory networks are rewired to accommodate perturbations. In addition, they reveal how iModulon gene membership may be corrupted by the use of non-wild-type samples, indicating a need for precaution while including non-wild-type samples in datasets intended for the study of wild-type regulation.

### iModulon-based graphical representation of the *E. coli* MG1655 TRN

**Figure 6.**
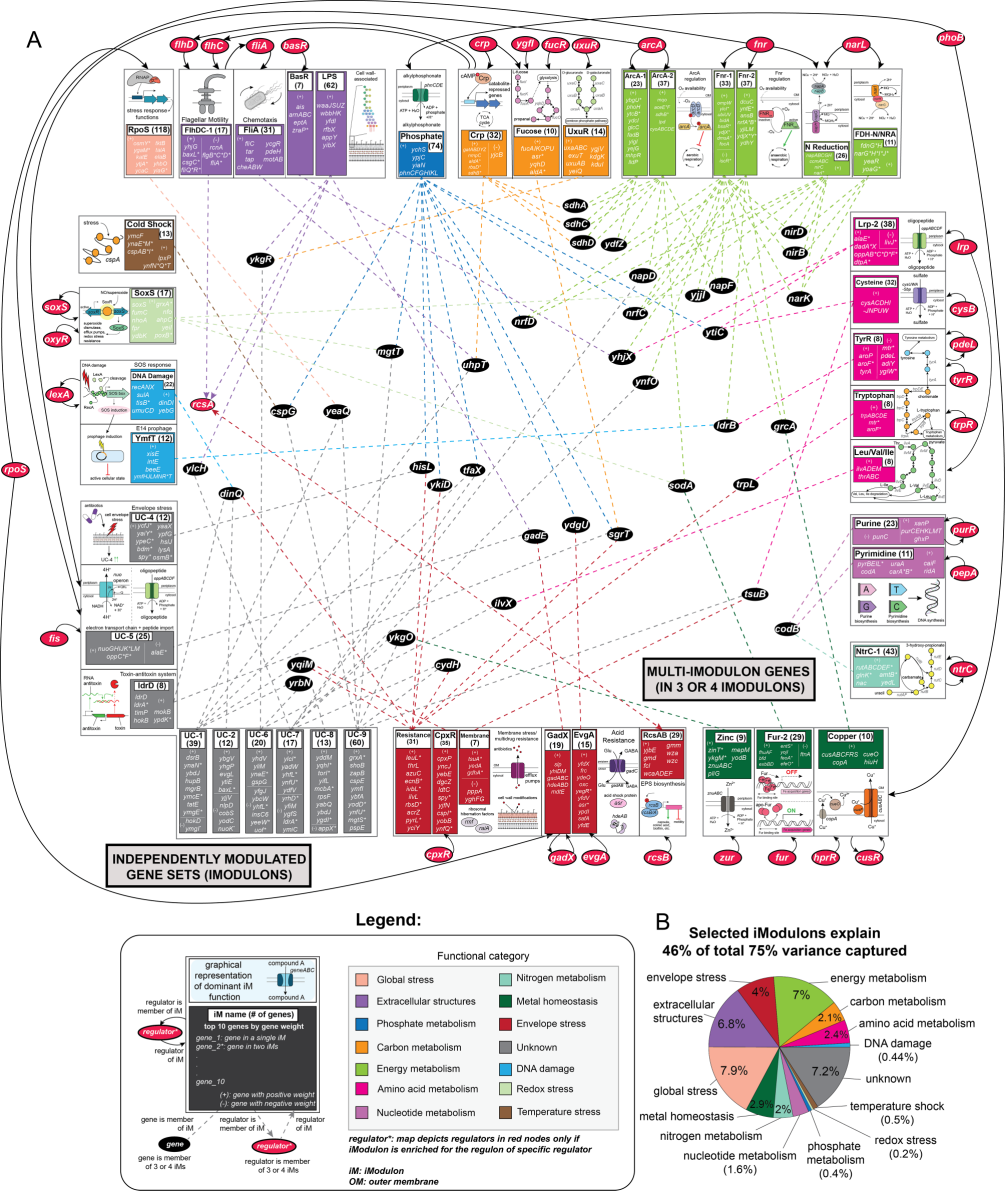
TRN map of *E. coli* MG1655. (**A**) A map of 45 selected iModulons in PRECISE-MG1655 [created in BioRender. Palsson, B. (2026) https://BioRender.com/ntae0u5]. iModulons containing at least one gene that is a member of three or four iModulons are depicted in the map as individual boxes. Each box is colored according to the functional category of the iModulon. Each characterized iModulon is accompanied by a graphical icon representative of its function. The top 10 genes in each iModulon based on absolute gene weight are listed within each box, along with (+) or (−) symbols to indicate positive and negative gene weights, respectively. A gene name followed by an asterisk is used to specify that the gene belongs to two iModulons totally. All genes found within three or four iModulons are displayed as black nodes at the center of the map, with dotted lines indicating which iModulons the gene belongs to. Dotted lines are colored based on the functional category of the iModulon that the gene belongs to. For iModulons with regulatory enrichment, regulators are depicted in red nodes if the iModulon is enriched for the regulon of the regulator. Note: For the purpose of clarity, iModulons categorized as “single gene” have been excluded. Additionally, only regulatory genes have been included as regulators in the map. The depiction of the regulator rpoD has been excluded for clarity. Detailed information on each iModulon can be found on iModulonDB.org. The map is also provided as a high-resolution figure in the Zenodo repository for the study. **(B)** Pie chart of the percentage of variance in gene expression that is explained by the selected 45 iModulons, with iModulons grouped by functional category.

The application of ICA to the PRECISE-MG1655 dataset thus generates a “purified” iModulon structure for *E. coli* MG1655, whose gene membership and activity state more closely align to that of the wild-type strain in comparison to those generated using previous PRECISE datasets. Our analysis leads to the construction of a multi-scale graphical representation of the TRN using iModulons (Fig. 6A). The iModulon-based map depicts a subset of 45 iModulons that represents the most interconnected portion of the TRN, identified based on iModulon gene membership. Specifically, all iModulons containing multi-iModulon genes present within three or four iModulons (i.e. 40 genes; Supplementary Table S11) are displayed in the map. Each characterized iModulon is accompanied by a graphical icon representing the associated biological function, and the iModulons are categorized based on their function. A list of highly weighted genes is provided for each iModulon, with a comprehensive list of iModulon gene members available on iModulonDB.org. Multi-iModulon genes present within three or four iModulons can be found at the center of the map. For regulatory iModulons, the map depicts regulator assignment as well as iModulon membership of regulators wherever applicable. Together, the map details how iModulons are linked to one another on the basis of three levels: functional categorization, gene membership, and regulation.

The map conveys multiple characteristics of the TRN that can be used to gain a deeper understanding of the network. For instance, we find that the 45 most interconnected iModulons explain over half of the total variance in gene expression explained by the iModulon structure as a whole (46% of total 75% variance) (Fig. 6B). iModulons within the map belonging to known functional categories like global stress, energy metabolism, and extracellular structures contribute to capturing the most variance in the dataset. Of the functional categories depicted, iModulons involved in energy metabolism are among the most interconnected iModulons, both through shared gene membership and regulation. This is likely due to the central role played by energy metabolism in the cell. We also note that several multi-iModulon genes belong to uncharacterized iModulons, with shared gene membership suggesting associations to other cellular functions such as envelope stress.

Overall, this iModulon-based map provides a pristine graphical representation of the most interconnected and complex networks within the *E. coli* MG1655 TRN. Future work aims to expand this preliminary version into a comprehensive map of the TRN using iModulons.

## Discussion

In this study, we presented a single-strain transcriptomic knowledgebase for wild-type *E. coli* MG1655. The expression component of PRECISE-MG1655 consists of 584 high-quality samples generated using a single experimental protocol. Applying ICA to this dataset resulted in the extraction of 115 iModulons (regulatory component of PRECISE-MG1655), which account for 75% of variance in gene expression captured by the RNA-seq compendium. Of the 115 iModulons, 77 iModulons are linked to known regulatory effects, explaining 76% of the total variance explained by iModulons. To our knowledge, the iModulon structure derived from the PRECISE-MG1655 dataset represents the most comprehensive, top-down delineated TRN for a wild-type microbial strain.

Against an invariant genetic background, we demonstrated the value of the knowledge-enriched TRN of *E. coli* MG1655 through a detailed analysis of the **M** and **A** matrices. The **M** matrix reveals that iModulon gene membership is largely non-overlapping, with 82% of 1385 iModulon genes belonging to a single iModulon. The remaining 18% of iModulon genes belong to, at most, four iModulons, revealing an intricate underlying network structure with iModulons being linked through shared genes. Specifically, cellular respiration-associated iModulons were found to have a high number of multi-iModulon genes present in more than two iModulons. The **M** matrix thus highlights genes linked to multiple independent transcriptional signals, thereby showcasing the complexity of regulation of certain cellular functions.

The activation state of the iModulons across the conditions represented in the RNA-seq compendium is provided by the **A** matrix, which quantifies the response of an iModulon under all experimental conditions. We demonstrated that the correlation between iModulon activities (i.e. rows of **A**) can be driven by conditions within specific projects, or can represent global relationships across all projects. By analyzing the fear-greed and cellular respiration-associated stimulons, PRECISE-MG1655 aids in characterizing relationships between groups of iModulons activated in a coordinated manner. Conversely, we use the columns of **A** to identify conditions that elicit similar transcriptomic responses, thereby demonstrating a novel application of the **A** matrix. Such an approach may be useful in designing a minimal condition space for the transcriptomic analysis of bacterial species. Furthermore, while we find that most conditions cluster together in a project-specific manner, iModulons highlight unique trends within projects. In particular, our case study on the “SNPv2” project identifies cellular respiration and motility as the main cellular functions significantly impacted by supplementation with different carbon and nitrogen sources at specific concentrations. Together, the **A** matrix provides a unique lens to study and compare both transcriptional dynamics (rows of **A**) as well as the conditions that elicit transcriptional responses in the bacterium (columns of **A**).

Importantly, our study provides a crucial improvement to previous PRECISE knowledgebases through the purification of iModulon gene membership by only utilizing wild-type samples from a single strain. By comparing the iModulon structures obtained from the PRECISE-MG1655 and PRECISE-1K datasets, we discovered that gene membership of 61 iModulons of PRECISE-MG1655 are well correlated (Pearson’s *r* ≥ 0.5) with iModulons of PRECISE-1K despite the presence of samples of mixed genetic background in the PRECISE-1K dataset. These iModulons, representing functions such as carbon and amino acid metabolism, are clear signals within the transcriptome that are consistently detected by ICA. However, we found that 35 iModulons of PRECISE-1K without a match in PRECISE-MG1655 are associated with ALE or genetic alterations. The effects of genetic perturbations are also evident in the well-correlated iModulons. Comparison of well-correlated iModulons between PRECISE-1K and PRECISE-MG1655 reveals changes in gene membership caused by TRN adaptations to genetic perturbations. Our case studies on the UC-4 iModulons, Sugar Diacid iModulons, and SoxS iModulons extracted from both datasets reveal that genes unique to the PRECISE-1K iModulons often do not reflect native regulation. Rather, they are linked to TRN adaptations due to genetic perturbations, allowing us to gain further insight into the malleable nature of the TRN. Additionally, these case studies show that inclusion of samples with variable genetic backgrounds results in the inclusion of genes with low weights in iModulons, thereby corrupting iModulon gene membership. Altogether, PRECISE-MG1655 highlights the need for datasets containing only single-strain wild-type samples to facilitate studies on wild-type regulation in a strain of interest.

Our characterization and analysis of the “purified” iModulon structure uncovered using the PRECISE-MG1655 dataset culminates in the construction of an iModulon-based graphical representation of the *E. coli* MG1655 TRN. This graphical representation is a multi-scale map of 45 iModulons, capturing the most interconnected and complex networks within the TRN based on iModulon gene membership. It uncovers inter-iModulon relationships based on gene membership and regulation in the wild-type strain. We aim to further develop the TRN map to generate a comprehensive iModulon-based representation of the TRN.

While the application of ICA to the PRECISE-MG1655 dataset uncovers a detailed iModulon structure for the TRN of this model strain, the comprehensiveness of the iModulon structure is limited by the condition space of the dataset. We find that 68% of genes are not found within any iModulon, either due to lack of activation or lack of activation in a coordinated manner with other genes. In order to increase iModulon gene coverage, there is a need to expand the PRECISE-MG1655 dataset through the addition of new samples (Supplementary Note S9). Previous studies have improved iModulon structures by adding samples grown under new sets of diverse conditions, such as various antibiotic treatments [48, 49] and use of different media types and growth phases [50]. The expansion of these datasets has not only provided key biological insights, but have also resulted in the extraction of new iModulons, thereby improving iModulon gene coverage. Additionally, underexplored condition types, such as high cell density growth that has been found to cause novel shifts in the transcriptome [51], hold potential in activating new gene sets. Such diverse conditions may also aid in activating genes with no known activation conditions. However, these conditions are often difficult to access and implement within a laboratory setting. Nevertheless, we expect the addition of new diverse condition types to PRECISE-MG1655 to contribute toward building a comprehensive top-down delineation of the TRN.

Thus, the PRECISE-MG1655 knowledgebase is a valuable resource for studying transcriptional regulation in the model wild-type strain *E. coli* MG1655 at the genome-scale. It enables a detailed exploration of the TRN through iModulon analysis and serves as a basis for understanding how the strain accommodates perturbations by rewiring transcriptional regulation. Together, PRECISE-MG1655 establishes the foundation for a quantitative TRN for the widely used *E. coli* MG1655 and serves as a reference for building and analyzing similar knowledgebases for other bacterial strains.

## Supplementary Material

gkag059_Supplemental_Files

## Data Availability

All code and data (aside from raw RNA-seq files) used to generate the results in this paper can be found on Zenodo (https://doi.org/10.5281/zenodo.15665274). New RNA-seq data reported in this study are publicly available, with the accession numbers listed in Supplementary Table S10. The M matrix, A matrix, M-binarized matrix, and iModulon table are also available in Supplementary Table S3, Supplementary Table S4, Supplementary Table S5, and Supplementary Table S6, respectively. The general iModulon analysis pipeline can be found on Zenodo (https://doi.org/10.5281/zenodo.18238196).
