# Supplementary material for: The transcriptional regulatory network of the Escherichia coli MG1655 reference strain
Source: Nucleic Acids Res. 2026 Feb 2;54(3):gkag059. doi: 10.1093/nar/gkag059 (PMC12862375; doi:10.1093/nar/gkag059)
Supplement: gkag059_Supplemental_Files [file gkag059_supplemental_files.zip › REVISED_SI_PRECISE_MG1655.pdf]

Supplementary Information:

The transcriptional regulatory network of the *Escherichia coli* MG1655 reference strain

Heera Bajpe[1], Jongoh Shin[1], Ying Hefner[1], Richard Szubin[1], Jaemin Sung[1], Yuan Yuan[1], Bernhard O. Palsson[1,2,3,4,5*]

1. Department of Bioengineering, University of California San Diego, La Jolla, CA, 92093, USA
2. Department of Pediatrics, University of California San Diego, La Jolla, CA, 92093, USA
3. Bioinformatics and Systems Biology Program, University of California, San Diego, La Jolla, USA
4. Center for Microbiome Innovation, University of California San Diego, La Jolla, CA 92093, USA
5. Novo Nordisk Foundation Center for Biosustainability, 2800, Kongens Lyngby, Denmark

*Address correspondence to Bernhard O. Palsson: bpalsson@ucsd.edu

Supplementary Figures:

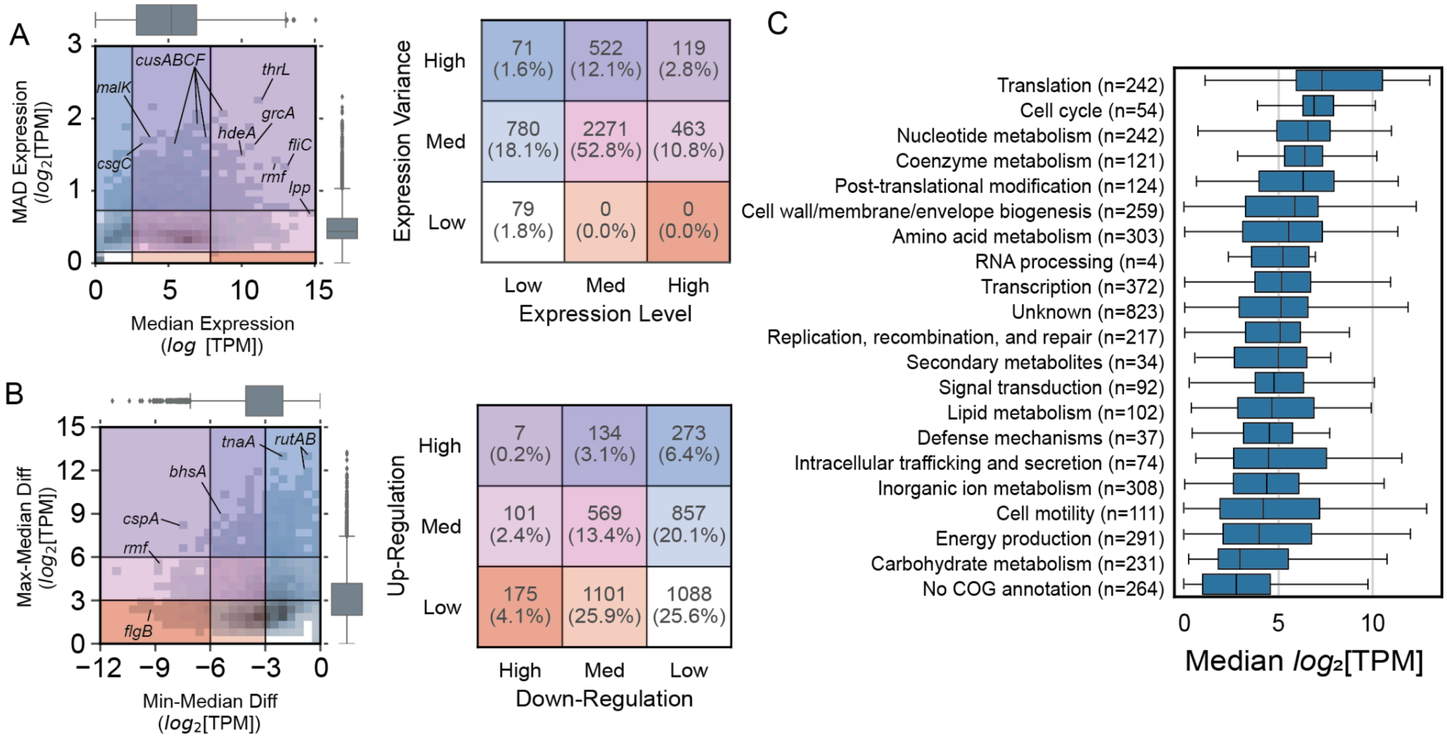

Supplementary Figure S1. Gene categorization based on expression and variability in expression. *(A) 2-D histogram of median gene expression plotted against median absolute deviation (MAD) of gene expression for all genes. The legend depicts the expression categories along with a gene count per category. The bounds for each category is defined at median ± 1 standard deviation. (B) 2-D histogram of difference between minimum and median expression plotted against the difference between maximum and median expression for all genes. The legend depicts the regulatory categories along with a gene count per category. The bounds for each category are defined as follows: low-to-medium split at 3 log_2[TPM] units (8-fold change from median expression); medium-to-high split at 6 log_2[TPM] units (32-fold change). (C) Median expression of gene categories based on cluster of orthologous group (COG) annotations.*

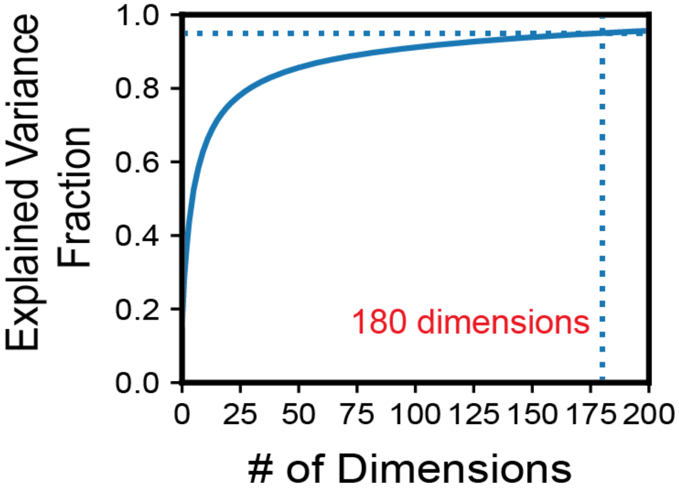

Supplementary Figure S2. *Cumulative explained variance plot depicting the total number of principal components required to explain 95% of variance in gene expression in the dataset.*

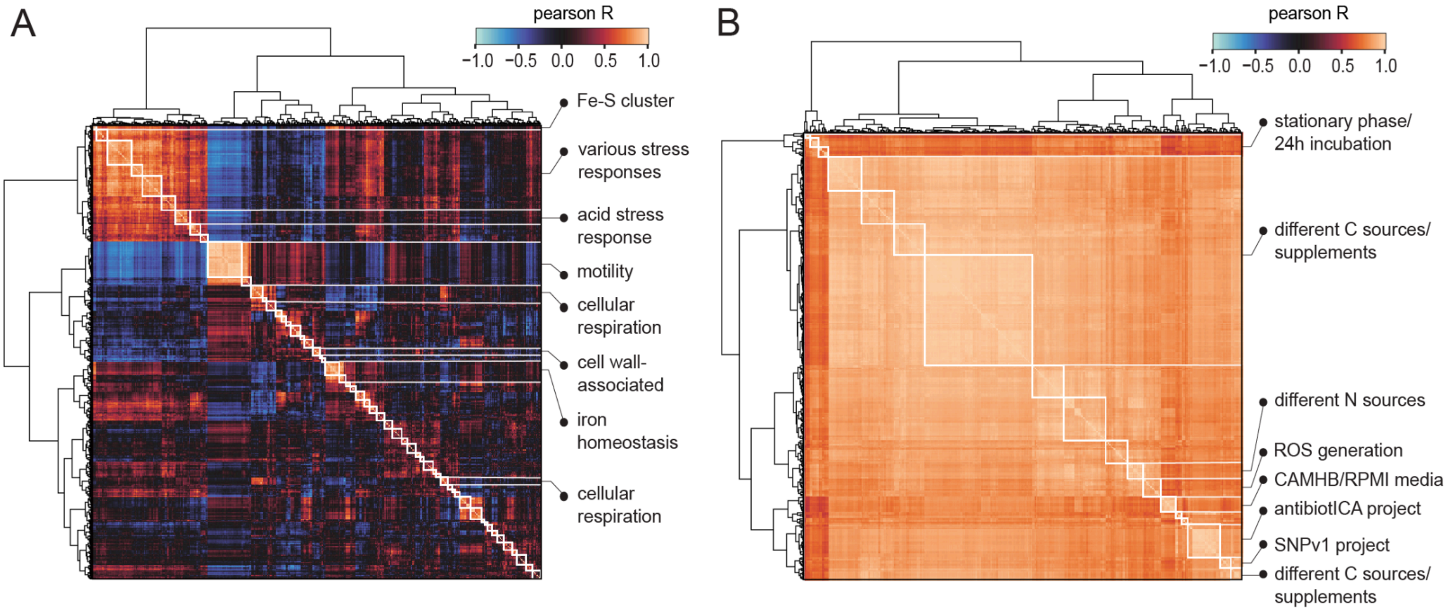

Supplementary Figure S3. Clustermaps of gene expression matrix. *(A) Clustermap of Pearson's r correlation of gene expression across all samples in the compendium for genes with high variability in expression. Variability in gene expression was calculated as the median absolute deviation across the dataset for each gene. Genes with high expression variability were defined as: expression variability > median expression variability across all genes + 1 standard deviation. (B) Clustermap of Pearson's r correlation of sample expression profiles for all samples in the compendium.*

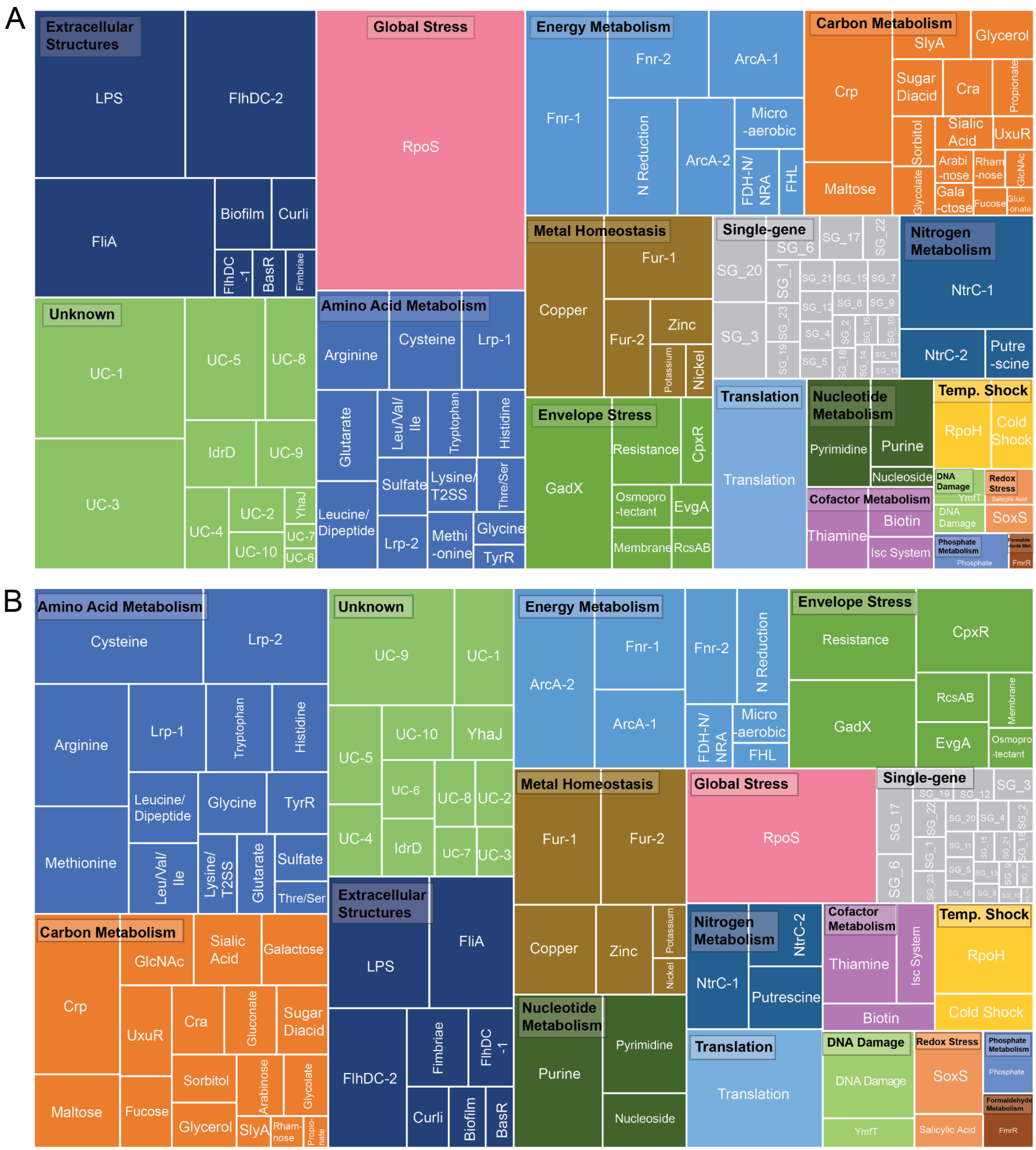

Supplementary Figure S4. Treemaps of the 115 iModulons extracted from the PRECISE-MG1655 dataset. *(A) The size of each rectangle represents the percentage of variance in gene expression that each iModulon explains. Together, the iModulons explain 75% of variance in gene expression in the dataset. (B) The size of each rectangle represents the mean weighted log_2[TPM] for the iModulon across project baseline conditions (See Supplementary Note S6). The color of each rectangle indicates the functional category of the iModulon.*

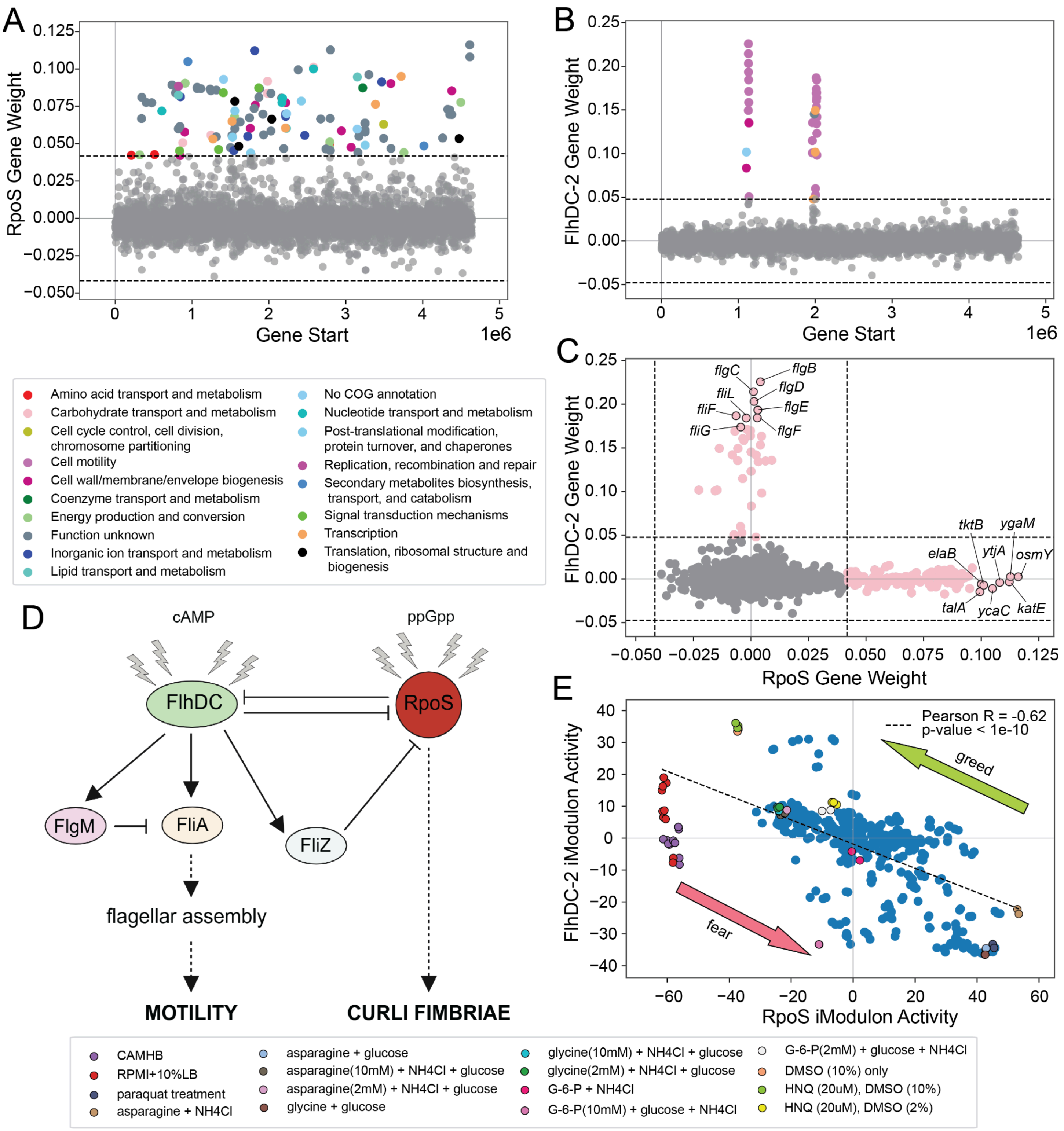

Supplementary Figure S5. iModulon case study. *(A-B) Gene membership of RpoS (A) and FlhDC-2 (B) iModulons. Each scatter plot depicts iModulon gene weights for all genes plotted against genomic position. COG categories for iModulon gene members are described in the legend. (C) Differential iModulon membership plot for the RpoS and FlhDC-2 iModulons. Dotted lines represent the gene weight threshold for each iModulon. Pink and gray dots are used to depict whether a gene is present in only one of the iModulons or does not belong to either iModulon, respectively. Highly weighted genes in each iModulon are labelled. (D) Simplified network of regulation of motility by FlhDC and RpoS. (E) Activity phase planes for RpoS and FlhDC-2 iModulons. Select conditions have been highlighted and described in the legend (G-6-P: glucose-6-phosphate).*

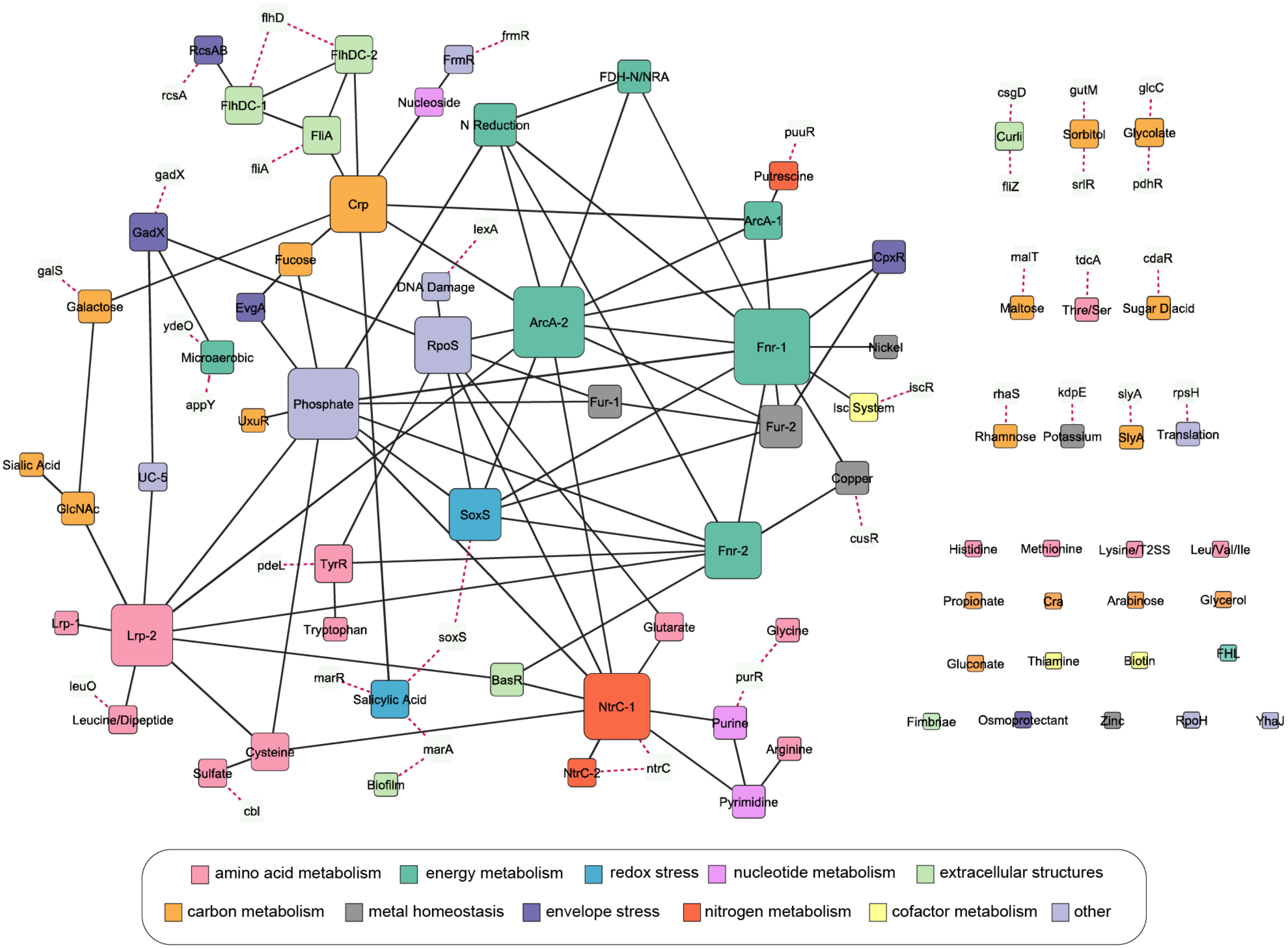

Supplementary Figure S6. *Network map of regulatory iModulons in PRECISE-MG1655. The 77 regulatory iModulons are represented by nodes, with each node being colored by functional category of the iModulon. Solid black edges between two iModulons indicate that they share at least one gene. Regulatory genes linked to iModulons through red dashed edges indicate that (1) the iModulon is enriched for the regulon of the regulator and (2) the regulator belongs to at least one iModulon. iModulon nodes are sized based on node degree.*

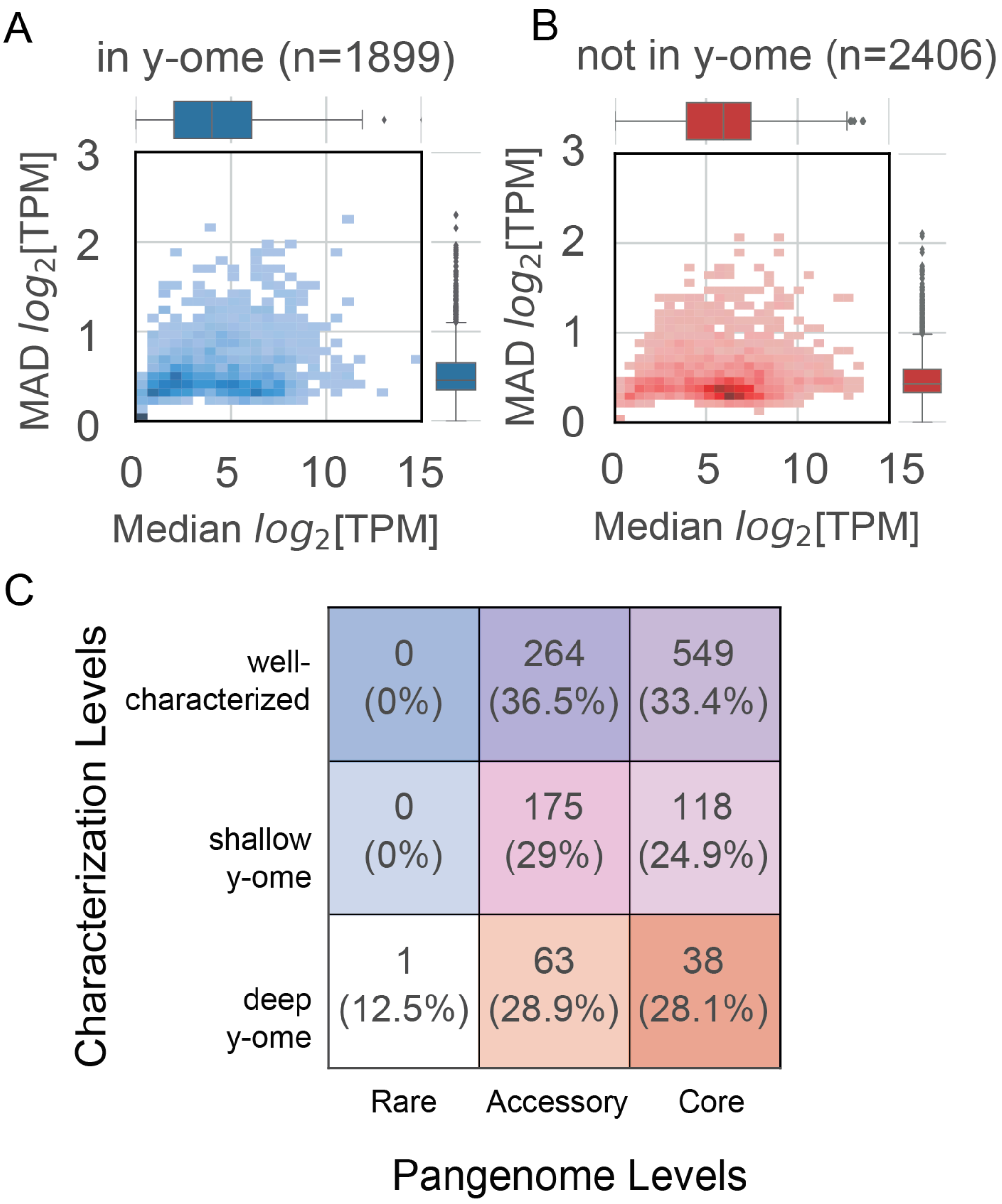

Supplementary Figure S7. Y-ome analysis. *(A,B) 2-D histogram of median gene expression vs median absolute deviation (MAD) of gene expression of poorly annotated genes (partial characterization or uncharacterized) (A) and well characterized genes (B), respectively. (C) iModulon genes categorized based on coverage of gene characterization and pangenome levels.*

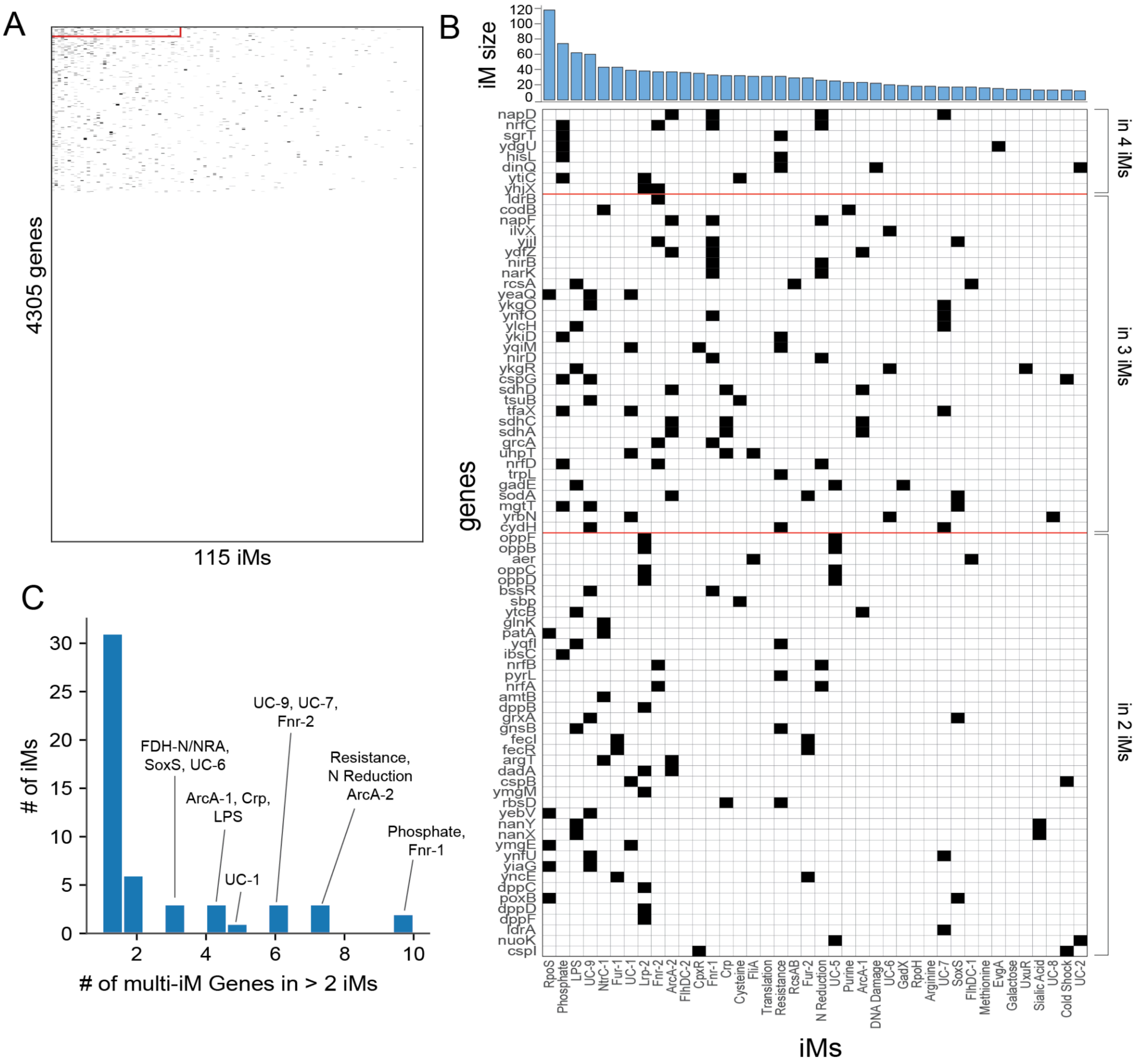

Supplementary Figure S8. Binarized M matrix. *(A) Plot of binarized M matrix. Black cells indicate that a gene belongs to the iModulon in the corresponding column. Both rows and columns are ordered by descending order of sum. Region in the red box represents the zoomed-in region depicted in Fig. 2F. (B) Higher resolution version of Fig. 2F representing top 10% of entries in gene presence/absence plot (40 iModulons/80 genes). Genes are sorted by iModulon membership count and iModulons are sorted by size in descending order. The bar chart represents the size of each iModulon. (C) Histogram of iModulon counts based on the count of multi-iModulon genes in more than two iModulons. (iM: iModulon)*

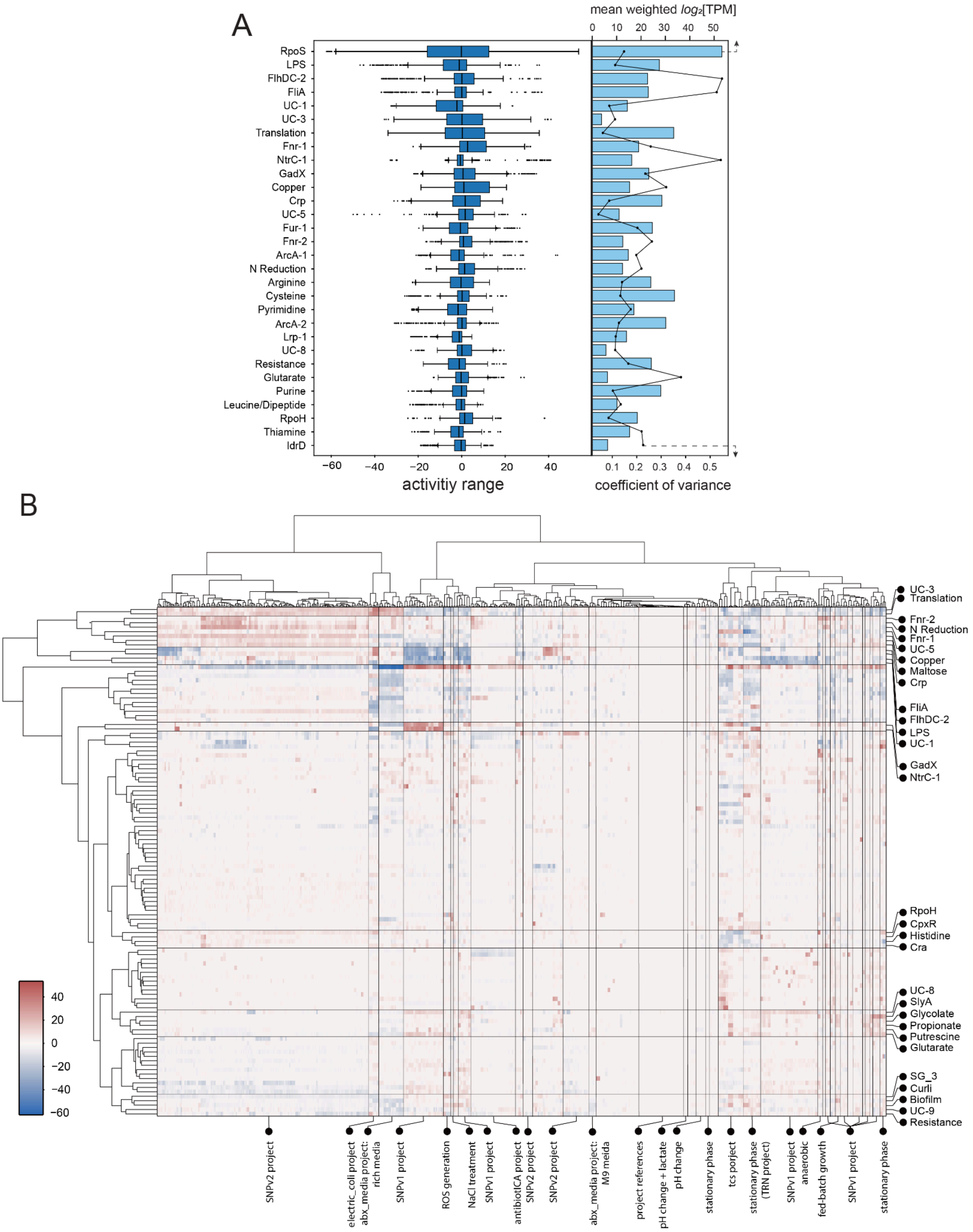
A
mean weighted log2[TPM]
0 10 20 30 40 50
RpoS
LPS
FlhDC-2
FliA
UC-1
UC-3
Translation
Fnr-1
NtrC-1
GadX
Copper
Crp
UC-5
Fur-1
Fnr-2
ArcA-1
N Reduction
Arginine
Cysteine
Pyrimidine
ArcA-2
Lrp-1
UC-8
Resistance
Glutarate
Purine
Leucine/Dipeptide
RpoH
Thiamine
IdrD
−60 −40 −20 0 20 40
activitiy range
0.1 0.2 0.3 0.4 0.5
coefficient of variance
B
UC-3
Translation
Fnr-2
N Reduction
Fnr-1
UC-5
Copper
Maltose
Crp
FliA
FlhDC-2
LPS
UC-1
GadX
NtrC-1
RpoH
CpxR
Histidine
Cra
UC-8
SlyA
Glycolate
Propionate
Putrescine
Glutarate
SG_3
Curli
Biofilm
UC-9
Resistance
40
20
0
−20
−40
−60
SNPv2 project
electric_coli project
abx_media project: rich media
SNPv1 project
ROS generation
NaCl treatment
SNPv1 project
antibiotICA project
SNPv2 project
SNPv2 project
abx_media project: M9 meida
project references
pH change + lactate
pH change
stationary phase
tcs porject
stationary phase (TRN project)
SNPv1 project
anaerobic
fed-batch growth
SNPv1 project
stationary phase

Supplementary Figure S9. Rows of the A matrix. *(A) Activity ranges of the top 30 iModulons in PRECISE-MG1655 based on explained variance. iModulons are ordered by explained variance in descending order. Bar plot depicts the mean weighted* log_2*[TPM] for each iModulon in project reference conditions (see Supplementary Note S6). Line plot displays the coefficient of variance for the weighted* log_2*[TPM] of each iModulon across the project reference conditions. (B) Clustermap of A matrix. Rows are labelled with iModulon clusters of interest. Columns are labelled by dominant project in each cluster or by condition type.*

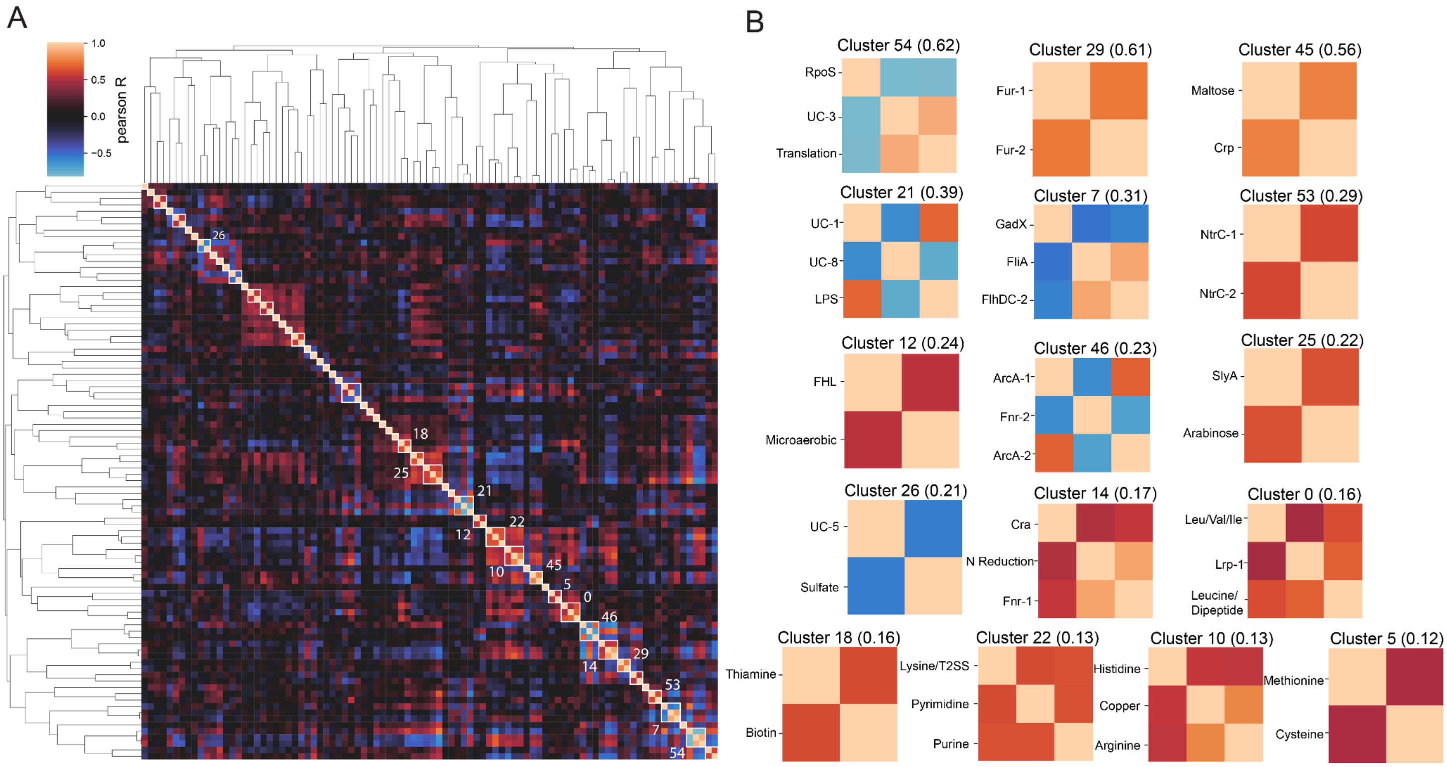

Supplementary Figure S10. *(A) Clustermap of Pearson's r correlation of iModulon activities (all non-technical iModulons) across all samples in compendium. (B) Best clusters obtained from clustermap of correlation of iModulon activities across all samples in compendium. Silhouette scores are listed in brackets.*

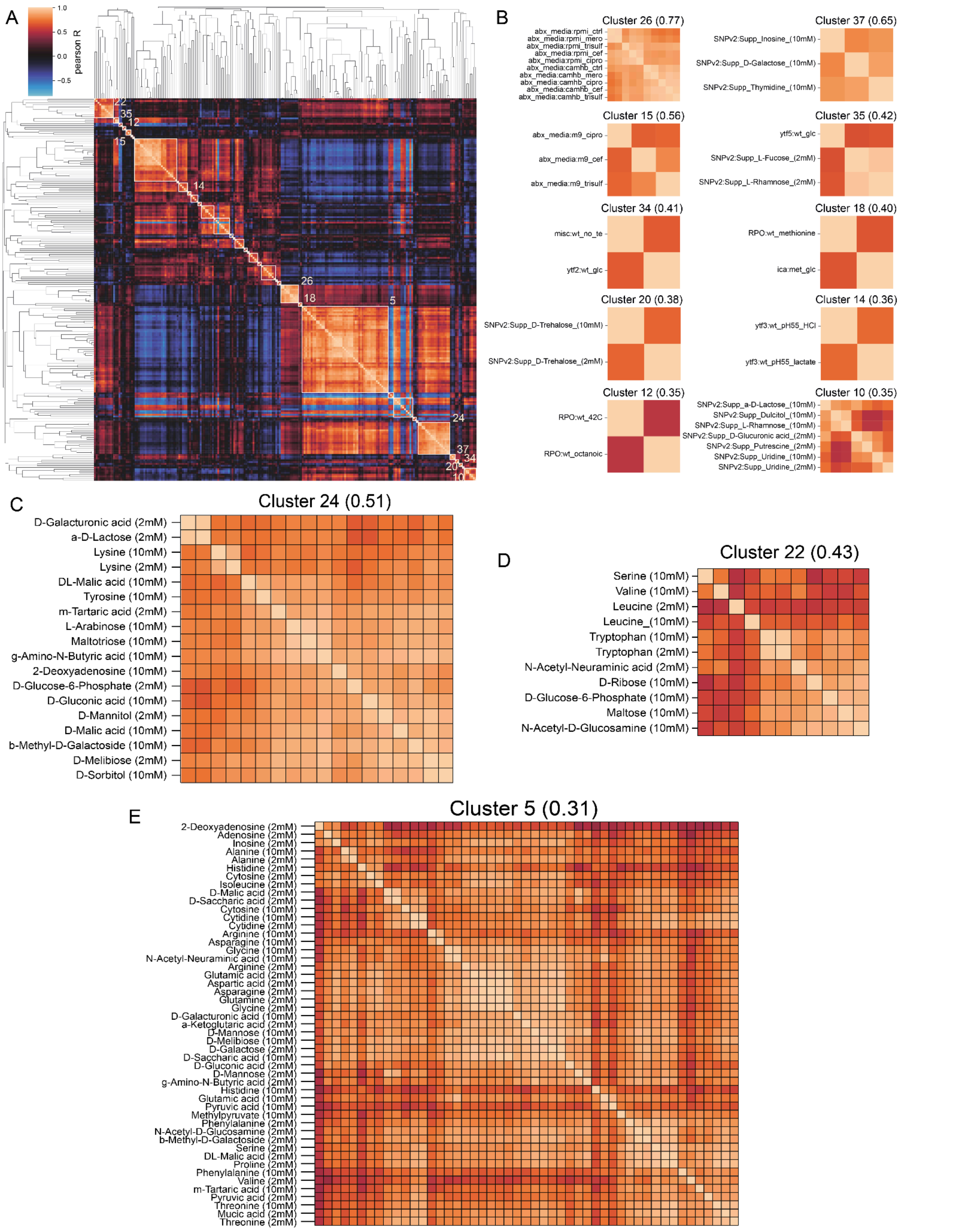

Supplementary Figure S11. *(A) Clustermap of Pearson's r correlation of sample activity profiles for select samples. Mean iModulon activity across replicates was used. (B-E) Best clusters (silhouette score > 0.3; listed in brackets) obtained from clustermap of sample activity profile correlation for select samples. Samples are labeled as "project_name:sample_id". Clusters 24, 22, and 5 are specific to the "SNPv2" project.*

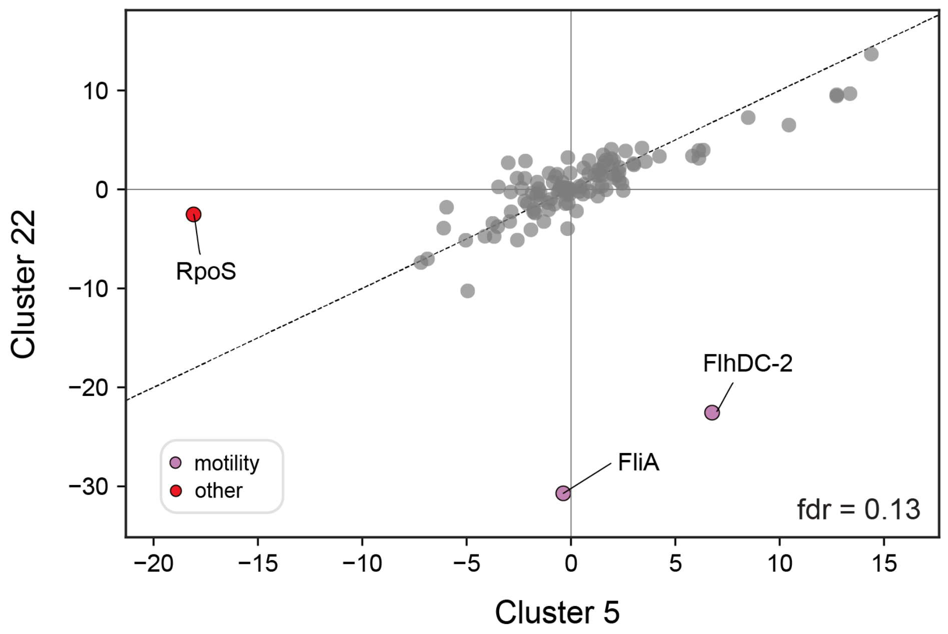

Supplementary Figure S12. *Differential iModulon activity plot for samples in Cluster 5 vs Cluster 22. Differentially activated iModulons are colored based on their functional category. Samples in each cluster are listed in Supplementary Fig. S11E and Supplementary Fig. S11D, respectively.*

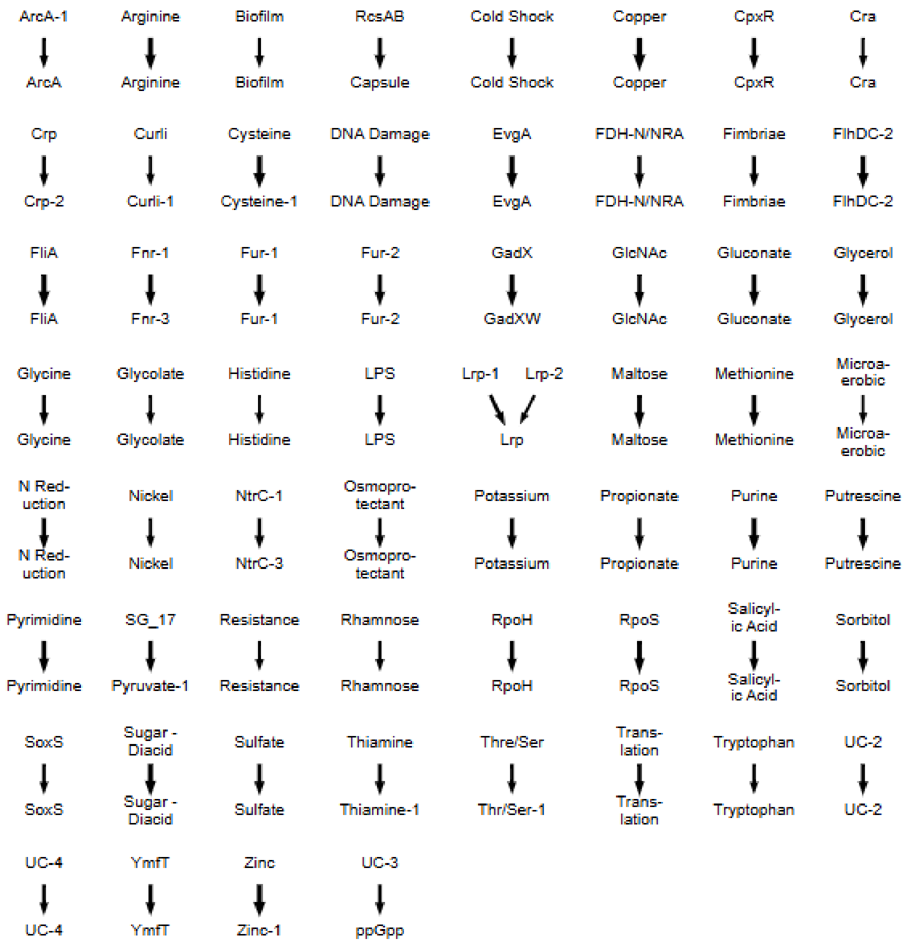

Supplementary Figure S13. *Mapping of PRECISE-MG1655 iModulons to PRECISE-1K iModulons (Pearson's r > 0.5).*

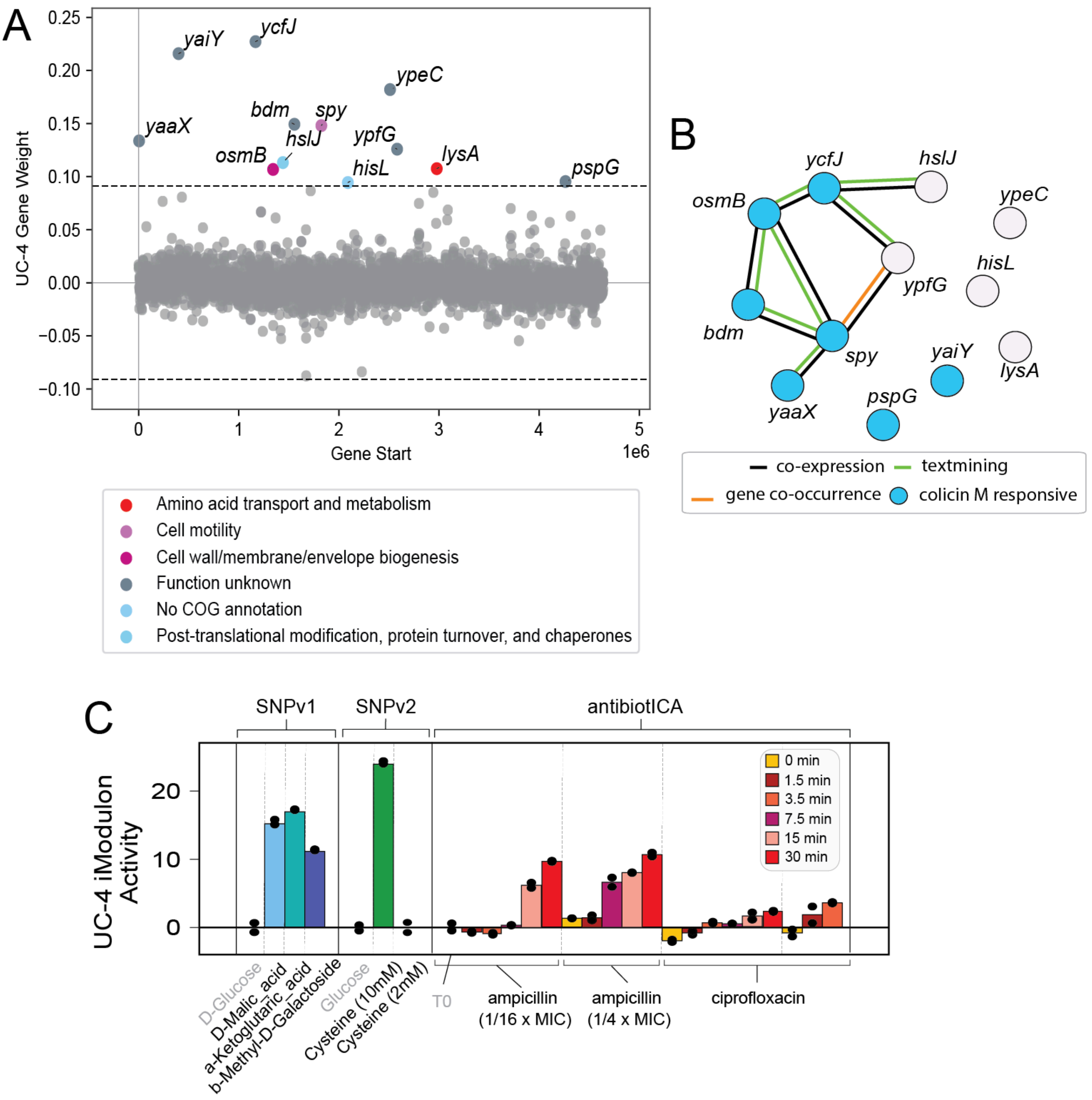

Supplementary Figure S14. Characterizing the UC-4 iModulon. *(A) Scatterplot of genomic position of all genes plotted against gene weight in the UC-4 iModulon of PRECISE-MG1655. (B) StringDB output for gene members of the PRECISE-MG1655 UC-4 iModulon. Genes previously found to be responsive to bacteriocin colicin M are colored in blue. (C) Activity of the UC-4 iModulon in PRECISE-MG1655 in select conditions. Project names are indicated above the plot, and baseline conditions for each project are labelled in grey.*

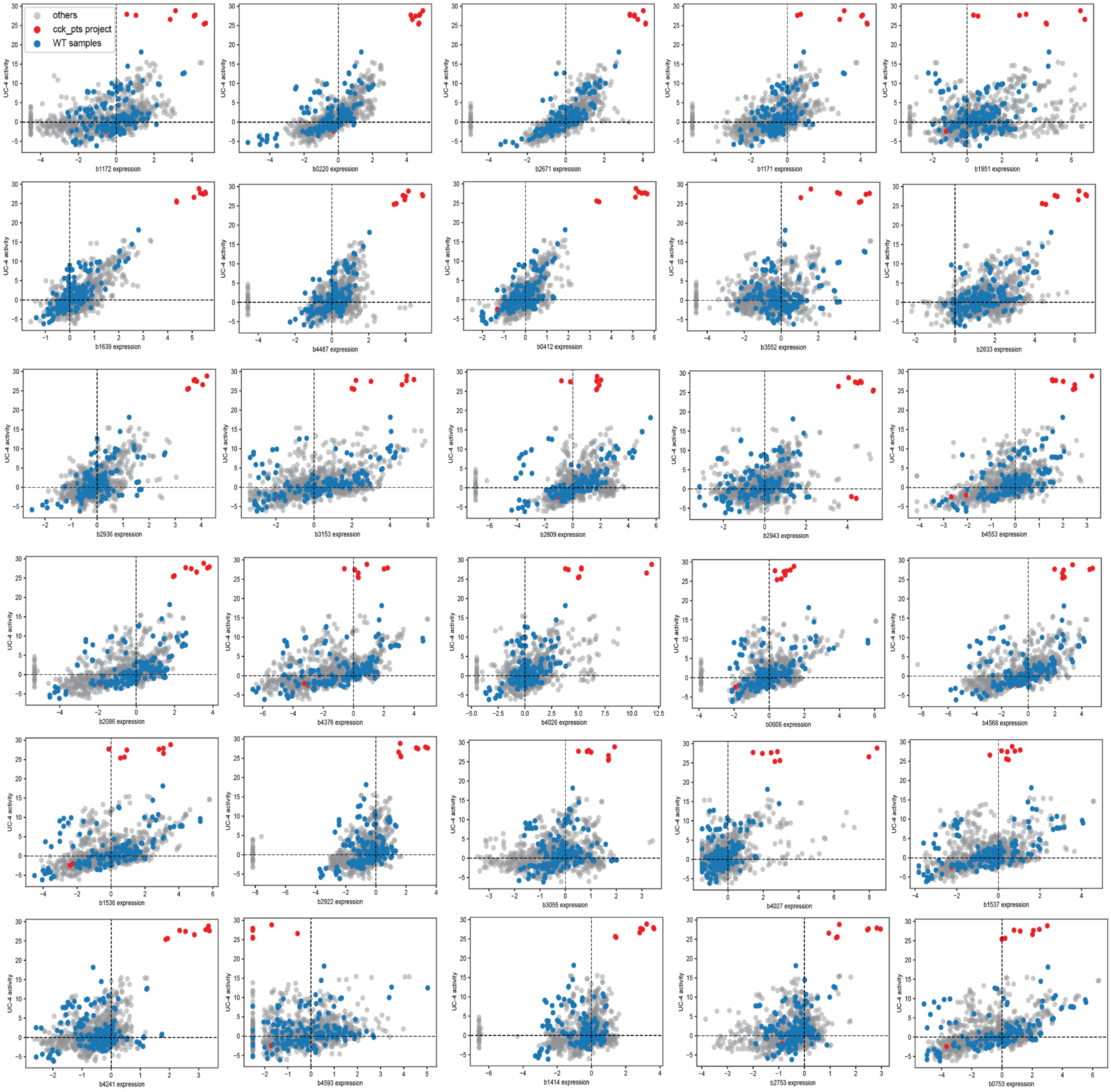

Supplementary Figure S15. *Scatterplots comparing centered gene expression for all genes unique to the UC-4 iModulon in the PRECISE-1K dataset to the activity of the PRECISE-1K UC-4 iModulon. Genes are ordered by descending order of gene weight in the UC-4 iModulon. (WT: wild-type)*

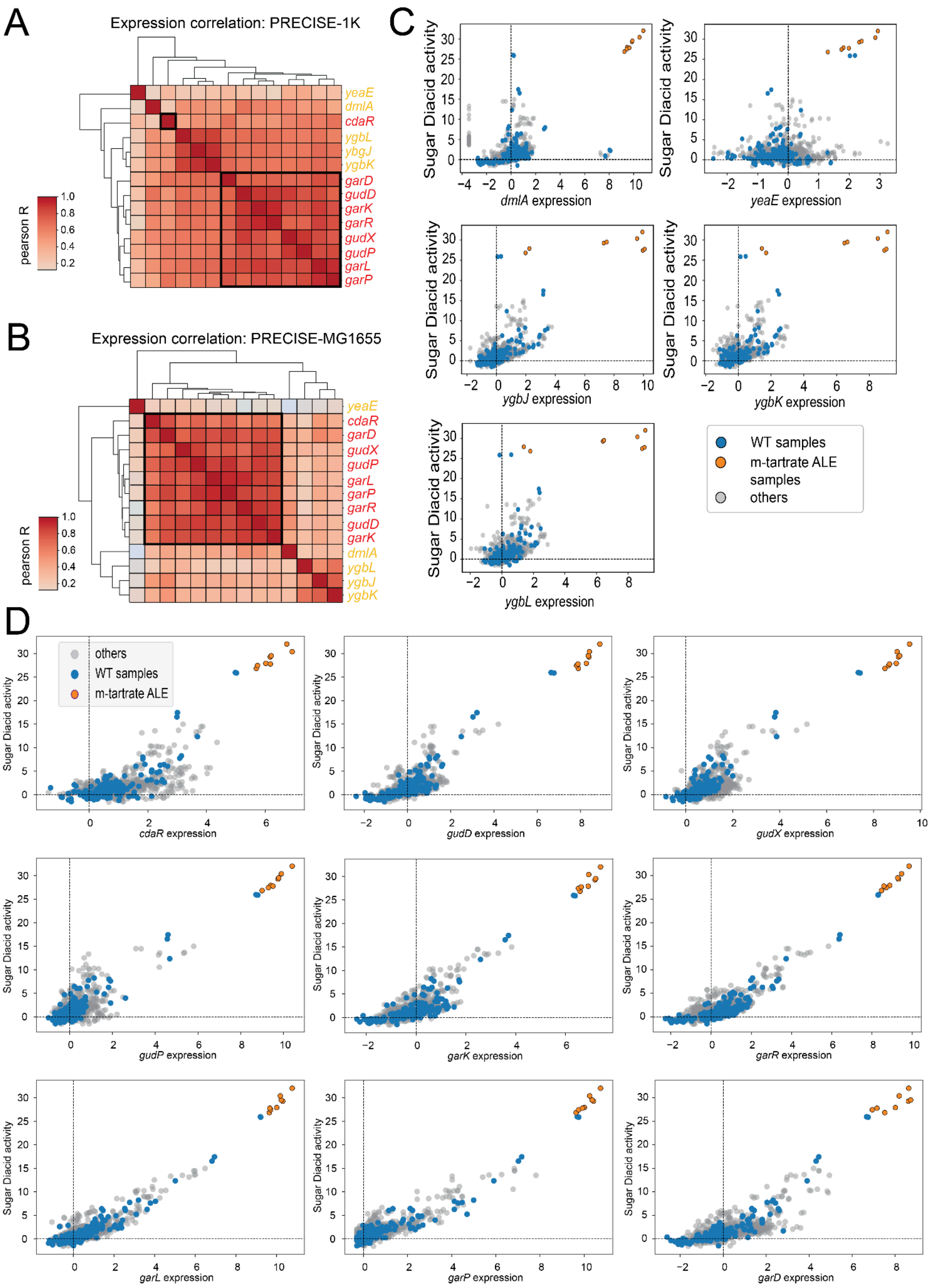
A
Expression correlation: PRECISE-1K
yeaE
dmlA
cdaR
ygbL
ybgJ
ygbK
garD
gudD
garK
garR
gudX
gudP
garL
garP
pearson R
B
Expression correlation: PRECISE-MG1655
yeaE
cdaR
garD
gudX
gudP
garL
garP
garR
gudD
garK
dmlA
ygbL
ygbJ
ygbK
C
Sugar Diacid activity
dmlA expression
yeaE expression
ygbJ expression
ygbK expression
ygbL expression
WT samples
m-tartrate ALE samples
others
D
others
WT samples
m-tartrate ALE
cdaR expression
gudD expression
gudX expression
gudP expression
garK expression
garR expression
garL expression
garP expression
garD expression

Supplementary Figure S16. Analysis of the unique genes of the PRECISE-1K Sugar Diacid iModulon. *(A) Clustermap of raw expression correlation (Pearson's r) of PRECISE-1K Sugar Diacid iModulon gene members in the PRECISE-1K dataset (red text: shared genes between Sugar Diacid iModulons of PRECISE-1K and PRECISE-MG1655; yellow text: unique genes in the PRECISE-1K Sugar Diacid iModulon). The black boxed regions highlight gene members present in the Sugar Diacid iModulon from both datasets. (B) Clustermap of raw expression correlation (Pearson's r) of PRECISE-1K Sugar Diacid iModulon gene members in the PRECISE-MG1655 dataset (red text: shared genes between Sugar Diacid iModulons of PRECISE-1K and PRECISE-MG1655; yellow text: unique genes in the PRECISE-1K Sugar Diacid iModulon). The black boxed region highlights gene members present in the Sugar Diacid iModulon from both datasets. (C) Scatter plots comparing centered gene expression for all genes unique to the PRECISE-1K Sugar Diacid iModulon to Sugar Diacid iModulon activity in the PRECISE-1K dataset. (D) Scatterplots comparing centered gene expression in the PRECISE-1K dataset for all genes shared by the Sugar Diacid iModulons in PRECISE-1K and PRECISE-MG1655 to the Sugar Diacid iModulon activity in the PRECISE-1K dataset. (WT: wild-type; ALE: adaptive laboratory evolution)*

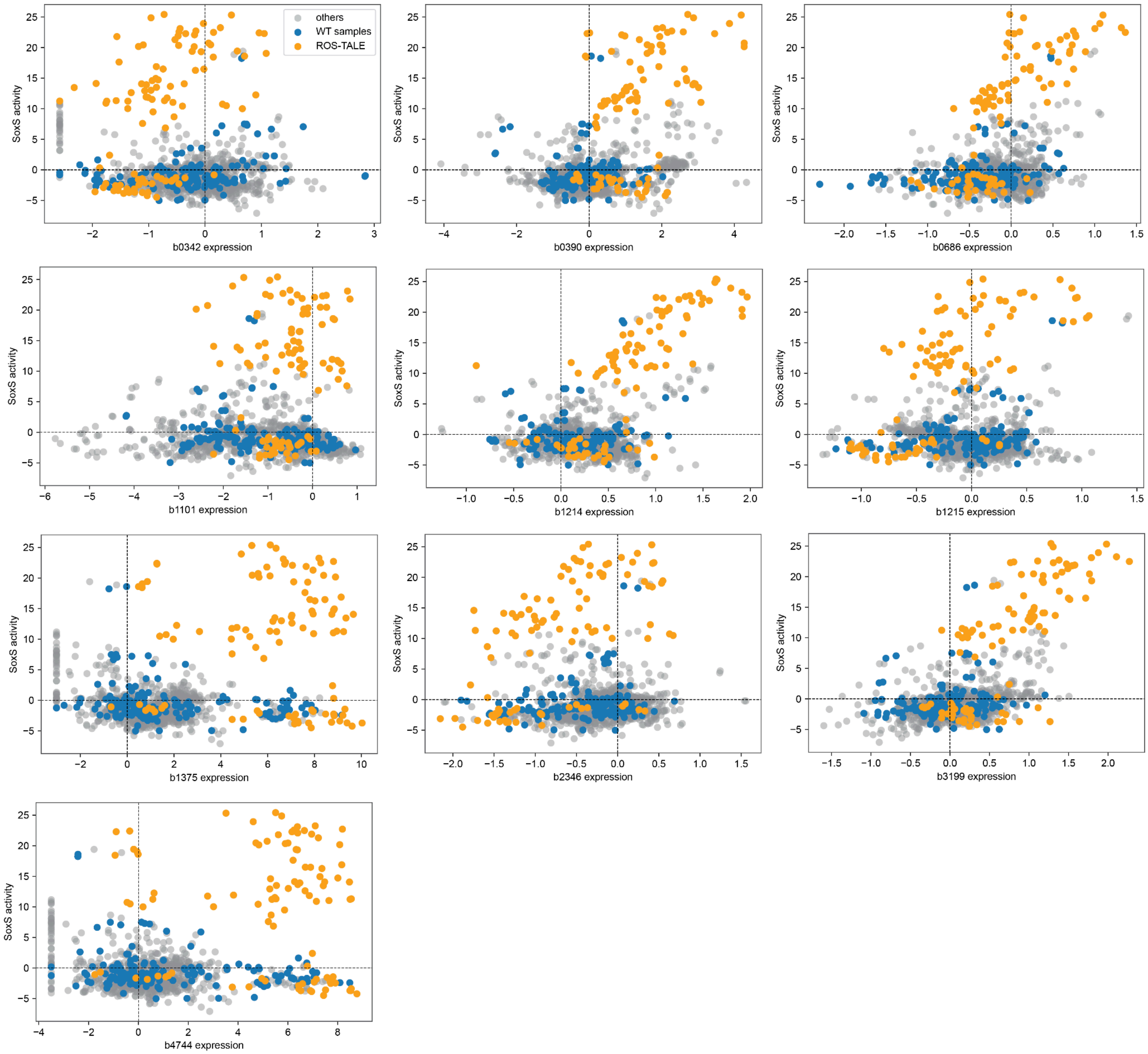

Supplementary Figure S17. *Scatterplots comparing centered gene expression for select genes unique to the SoxS iModulon in the PRECISE-1K dataset to the activity of the PRECISE-1K SoxS iModulon. The top ten genes with absolute*

gene weights closest to the threshold of the SoxS iModulon (threshold = 0.03) were selected (all genes with weights < 0.032 in the SoxS iModulon). (WT: wild-type)

Supplementary Notes:

S1. Data reuse in PRECISE-MG1655

In addition to 584 unique RNA-seq samples, the dataset to which ICA was applied contains two samples reused from a previous study in PRECISE-1K. Specifically, the ‘wt_Fe’ samples in the ‘CFP’ project were reused from the ‘fur’ project. The original ‘wt_Fe’ samples from the ‘fur’ project serve as the reference condition for the project, and were used as the baseline condition to normalize the $\log_2$[TPM] data for the two other samples in the project. On the other hand, the reused ‘wt_Fe’ samples from the single-condition “CFP” project are normalized to the reference condition of the “control” project (See Methods). Reusing this data with varying normalization methods allows ICA to identify different signals from a single condition.

S2. Gene categorization based on expression and variation

We have previously demonstrated the ability of the PRECISE-1K database to describe global trends associated with genome-scale gene expression, variation, and regulation(1). Here, we applied a similar approach to leverage the diverse range of conditions in the single-strain PRECISE-MG1655 dataset to categorize genes based on their wild-type expression and variation in expression. First, median gene expression was compared to median absolute deviation (MAD) of expression for all genes. We used MAD to determine gene expression variability due to its robustness to outliers that are observed in gene expression data. Using the distribution thus obtained, we categorized genes based on their expression level and variation (Supplementary Fig. S1A). As previously found using PRECISE-1K, the protein with the highest abundance in *E. coli*, *lpp* (encoding murein lipoprotein), exhibits the highest median expression and a moderate level of variation(2, 3). This category of highly expressed genes with medium variation also includes other genes that are crucial to the maintenance of stability and growth of the bacteria, such as genes encoding outer membrane proteins and ribosomal subunits. Genes with medium to high expression accompanied by high variation appear to be involved in specific stress responses such as acid stress (*hdeA*) or copper stress (*cusABCF*), as well as motility (ex: *fliC*). Additionally, our findings reflect those of PRECISE-1K, with more than 50% of genes having both medium expression and variation. Furthermore, the gene category having both low expression and variation largely consists of prophage genes, insertion sequences, and uncharacterized genes.

Next, we studied the influence of regulation on gene expression by assessing the range of expression of each gene (Supplementary Fig. S1B). We found that 74.5% of genes showcase at least medium upregulation or downregulation in the dataset, indicating the extent of the effect of regulation on gene expression. Some genes exhibited relatively larger effects in a single direction. For example, the flagellar gene *flgB* shows a narrow range of expression in the positive direction. However, this gene exhibits large effects in the negative direction, with strong downregulation observed in stationary phase samples. Genes with both high upregulation and downregulation include *cspA*, whose wide range of expression levels likely contribute to stress response and efficient translation(4, 5). Overall, genes belonging to the functional categories ‘Cell Cycle’ and ‘RNA Processing’ have a more narrow range of inducibility in comparison to categories such as ‘Translation’, ‘Motility’, and ‘Cell wall/membrane/envelope biogenesis’ (Supplementary Fig. S1C). Additionally, the expression of genes belonging to categories essential for the basic functioning of cells, such as ‘Translation’, ‘Cell Cycle’, and ‘Nucleotide Metabolism,’ have a higher median expression compared to other categories.

The above findings demonstrate the utility of the PRECISE-MG1655 dataset in enabling the exploration of wild-type gene expression at the genome scale. It highlights both expected and novel trends in gene expression and variation, as well as its potential in mining for genome-wide regulatory signatures.

S3. Clustering of gene expression matrix indicates underlying structure within data

Of the 4,305 genes with detected expression, we clustered gene expression correlations for all genes with high variability in expression to reveal underlying patterns in gene expression (Supplementary Fig. S3A). Variability in gene expression was calculated as the MAD across the dataset for each gene. As stated in Supplementary Note S2, the MAD was selected due to its robustness to outliers. Genes with high expression variability were defined as: expression variability > median expression variability across all genes + 1 standard deviation. Among the identified clusters, several clusters containing genes involved in stress responses were found, such as oxidative and acid stress. Additionally, many clusters contained genes with shared regulators; for instance, the two clusters associated with iron homeostasis mostly consist of genes regulated by the global transcription factor Fur, possibly revealing patterns within the Fur regulon. Furthermore, two motility-associated clusters with strong negative correlations with the stress response clusters were identified, indicating a trade-off between the expression of motility and stress mitigation genes.

Clustering the correlation of expression profiles of all samples across the compendium revealed that the transcriptome composition across conditions is quite similar (Supplementary Fig. S3B). While some clusters contained samples generated from a single project, several clusters highlighted condition types that result in similar transcriptomic responses in *E. coli* MG1655. Most stationary phase samples clustered together and were more weakly correlated with the rest of the samples in the compendium. Clustering also revealed patterns within transcriptomic responses elicited using different carbon and nitrogen sources/supplements as well as media types.

S4. Case Study: Analysis of RpoS and FlhDC-2 iModulons

The RpoS iModulon is the primary stress response iModulon of *E. coli* MG1655, enriched for the regulon of the sigma factor RpoS. Highly weighted genes in this iModulon include *osmY*, *katE*, *ygmA*, and *tktB*, which are involved in the response to different stresses, such as osmotic stress, oxidative stress, and stationary phase growth. The functions of the gene members of the RpoS iModulon, as well as their scattered genomic positions, indicate that the iModulon represents a broad cellular response to environmental stress (Supplementary Fig. S5A). The FlhDC-2 iModulon represents genes involved in flagellar assembly, enriched for the regulon of the master regulator FlhDC. A majority of the genes belonging to this iModulon can be found in two regions of the genome, and encode proteins forming structural parts of the flagella (Supplementary Fig. S5B). Highly weighted genes in the iModulon include *flgBDCE*, involved in the formation of the hook and rod of the flagella.

Considering the distinct functions of the RpoS and FlhDC-2 iModulons, it is expected that they have distinct gene members (Supplementary Fig. S5C). However, it is known that the regulators for which the iModulons are enriched for, RpoS and FlhDC, exert inhibitory effects on one another (Supplementary Fig. S5D)(6). The resultant effect can be observed through the negative correlation between the activities of the RpoS and FlhDC-2 iModulons, indicating a trade-off between motility and stress responses (Supplementary Fig. S5E). The activity phase plane shows that in conditions eliciting fear, such as paraquat treatment, the RpoS iModulon is upregulated and the FlhDC-2 iModulon is downregulated. In contrast, most samples grown in rich media exhibit a more greedy behaviour, with low RpoS iModulon activity and high FlhDC-2 iModulon activity.

The phase plane also reveals how motility and stress are fine-tuned based on nutrient availability in the environment. With the use of poor nutrient sources such as amino acids (ex: asparagine and glycine) for carbon or nitrogen sources, the bacterium exhibits a fearful response; the RpoS iModulon is upregulated, while the FlhDC-2 iModulon is downregulated. On the other hand, supplementation of these amino acids results in a greedy response; the FlhDC-2 iModulon is upregulated whereas the RpoS iModulon is downregulated. A similar trend can be observed in the presence of complex sugars such as glucose-6-phosphate. However, the motility of the bacterium appears to depend on the concentration of glucose-6-phosphate present in the media; while supplementation at low concentrations (2 mM) upregulates motility as expected, higher concentrations (10 mM) downregulate motility. This may be due to the abundance of more favourable nutrients in the media, thereby reducing the need for foraging.

Hence, this case study highlights that the iModulons extracted from PRECISE-MG1655 represent clear biological signals in the *E. coli* MG1655 transcriptome. Overall, the analysis of these iModulons facilitates rapid characterization of the TRN.

S5. Precision and recall calculations on iModulonDB

Precision and recall values for the iModulons extracted from the PRECISE-MG1655 dataset were calculated through the expanded regulatory enrichment method described by Lamoureux et al.(1), which considers evidence levels for regulatory interactions before determining regulon gene membership. These values can be found in the iModulon table (Supplementary Table S6), on the PRECISE-MG1655 main dataset page on iModulonDB, and the iModulon summary box on each iModulon page of iModulonDB.

The calculations performed in the “Regulon Overlap” section on each iModulon page of iModulonDB do not utilize evidence levels for regulatory interactions. Rather, they consider all genes regulated by a certain regulator as part of the regulon of that regulator, regardless of evidence levels. For the purpose of consistency across the website, regulon gene membership in the “Regulon Overlap” section of PRECISE-MG1655 iModulons was also calculated in the same manner. Hence, the values present in this section may differ from the values provided in the iModulon table.

S6. Rows of the A matrix: activity ranges of iModulons

To quantify the zero-activity state of an iModulon, we calculated a mean weighted $\log_2$[TPM] value for each iModulon across all reference conditions. This was done by first determining the weighted $\log_2$[TPM] value for a single reference condition sample by summing the weighted expression of all genes in an iModulon, and subsequently averaging across all replicates for that reference condition. This value was determined for all conditions in the compendium used as a reference state while centering the $\log_2$[TPM] data, and then averaged to specify a single weighted $\log_2$[TPM] value for an iModulon across all reference states.

Functional categories such as motility were identified as variable across the baseline conditions, while others were less variable, as previously observed (Supplementary Fig. S9A)(1). The median range of activity around the referenced (i.e., zero) activity state for the 115 iModulons in PRECISE-MG1655 is 35.5, while the GlcNAc and RpoS iModulons display the highest (115.16) and lowest (20.16) activity ranges for non-technical iModulons, respectively. Additionally, the RpoS iModulon exhibits the highest level of upregulation and downregulation. Overall, iModulons linked to global regulators, such as the RpoS, Translation, and FliA iModulons, tend to have larger ranges of activities, whereas those involved in more specialized functions such as carbon metabolism (GlcNAc, Glycolate, and Maltose iModulons) have smaller ranges of activities.

S7. Characterization of the UC-4 iModulon

The twelve genes in the UC-4 iModulon of PRECISE-MG1655 are spread across the genome, with eight genes having unknown functions (Supplementary Fig. S14A). However, this iModulon is likely a biological signal, considering the presence of a similar iModulon in PRECISE-1K. Additionally, several genes in this iModulon have previously been found to be responsive to the antibiotic colicin M, known to inhibit cell wall synthesis (Supplementary Fig. S14B)(7). In agreement with these findings, we observed that the iModulon is upregulated during treatment with malic acid, which is also known to cause cell envelope stress (Supplementary Fig. S14C)(8). Additionally, treatment with ampicillin, also targeting cell wall synthesis, was observed to upregulate the UC-4 iModulon at later time points. These findings indicate that the UC-4 iModulon is involved in the response to cell envelope stress. We can thus hypothesize that other supplements that upregulate the iModulon, such as α-ketoglutarate, methyl β-D-galactoside, and excess cysteine, may trigger a cell envelope stress response in *E. coli* MG1655.

S8. Unique genes in the Sugar Diacid iModulon of PRECISE-1K

The Sugar Diacid iModulon of PRECISE-1K contains nine shared genes and five unique genes in comparison to that of PRECISE-MG1655. While there does not appear to be a clear functional link between the set of shared and unique genes, the expression of the unique genes is notably more closely correlated to the shared genes in PRECISE-1K compared to that in PRECISE-MG1655 (Supplementary Fig. S16A-B). We plotted the expression of the Sugar Diacid iModulon genes against the activity of the Sugar Diacid iModulon in the PRECISE-1K dataset and found that the expression of the unique genes is strongly correlated with the activity of the Sugar Diacid iModulon in samples generated through the evolution of *E. coli* with m-tartrate. (project: "Enzyme Promiscuity"; Supplementary Fig. S16C-D)(9). This trend cannot be observed with the wild-type samples, thereby attributing the presence of the unique genes in the PRECISE-1K Sugar Diacid iModulon to evolution with m-tartrate.

S9. PRECISE-MG1655 dataset expansion

In order to generate a more comprehensive iModulon structure using the PRECISE-MG1655 dataset, there is a need to increase iModulon gene coverage by expanding the size of the dataset. While several condition types remain unexplored due to costs of data generation and difficulty in accessing within a laboratory setting, we can use the PRECISE-MG1655 and PRECISE-1K knowledgebases to inform condition space design for future studies. While several differences between the iModulon structures obtained from the two datasets are due to the presence of non-wildtype samples in PRECISE-1K (ex: "ALE Effects" and "Genetic Alterations" categories), some differences can be attributed to features of the condition-space that are not associated with the strain type. For example, some non-wildtype samples in the PRECISE-1K dataset include the addition of supplements that are not present in the PRECISE-MG1655 dataset. For instance, the PRECISE-1K dataset contains evolved samples supplemented with xylose (project: "substrate-switching evolution")(10). While the inclusion of these samples in the dataset contributed to the detection of non-wildtype regulatory signals by ICA, the supplementation of xylose in these samples resulted in the detection of the Xylose iModulon (containing *xylABEFGHR*; involved in xylose transport and catabolism). The *xyl* operon genes within the Xylose iModulon of PRECISE-1K are among the 2,920 genes that are not found within any iModulon of PRECISE-MG1655. Addition of a xylose supplementation condition to the PRECISE-MG1655 dataset will likely lead to the detection of a xylose transport and catabolism iModulon containing similar gene membership to that of the Xylose iModulon in PRECISE-1K. Similarly, such comparisons of the iModulon structures obtained from the two datasets will aid in the identification of other "easy to access" conditions that will address condition space design while increasing dataset size to improve iModulon gene coverage.

References:

1. Lamoureux CR, Decker KT, Sastry AV, Rychel K, Gao Y, McConn JL, Zielinski DC, Palsson BO. 2022. A multi-scale transcriptional regulatory network knowledge base for Escherichia coli. bioRxiv https://doi.org/10.1101/2021.04.08.439047.

2. Li G-W, Burkhardt D, Gross C, Weissman JS. 2014. Quantifying Absolute Protein Synthesis Rates Reveals Principles Underlying Allocation of Cellular Resources. Cell 157:624–635.

3. Braun V, Rehn K. 1969. Chemical Characterization, Spatial Distribution and Function of a Lipoprotein (Murein-Lipoprotein) of the E. coli Cell Wall. Eur J Biochem 10:426–438.

4. Bae W, Jones PG, Inouye M. 1997. CspA, the major cold shock protein of Escherichia coli, negatively regulates its own gene expression. J Bacteriol 179:7081–7088.

5. Delaleau M, Figueroa-Bossi N, Do TD, Kerboriou P, Eveno E, Bossi L, Boudvillain M. 2024. Rho-dependent transcriptional switches regulate the bacterial response to cold shock. Mol Cell 84:3482-3496.e7.

6. Mika F, Hengge R. 2013. Small Regulatory RNAs in the Control of Motility and Biofilm Formation in E. coli and Salmonella. Int J Mol Sci 14:4560–4579.

7. Kamenšek S, Žgur-Bertok D. 2013. Global transcriptional responses to the bacteriocin colicin M in Escherichia coli. BMC Microbiol 13:42.

8. Ji Q-Y, Wang W, Yan H, Qu H, Liu Y, Qian Y, Gu R. 2023. The Effect of Different Organic Acids and Their Combination on the Cell Barrier and Biofilm of Escherichia coli. 16. Foods 12:3011.

9. Guzmán GI, Sandberg TE, LaCroix RA, Nyerges Á, Papp H, Raad M de, King ZA, Hefner Y, Northen TR, Notebaart RA, Pál C, Palsson BO, Papp B, Feist AM. 2019. Enzyme promiscuity shapes adaptation to novel growth substrates. Mol Syst Biol https://doi.org/10.15252/msb.20188462.

10. Sandberg TE, Lloyd CJ, Palsson BO, Feist AM. 2017. Laboratory Evolution to Alternating Substrate Environments Yields Distinct Phenotypic and Genetic Adaptive Strategies. Appl Environ Microbiol

83:e00410-17.
